# Pre‐diagnostic blood immune markers, incidence and progression of B‐cell lymphoma and multiple myeloma: Univariate and functionally informed multivariate analyses

**DOI:** 10.1002/ijc.31536

**Published:** 2018-04-26

**Authors:** Roel Vermeulen, Fatemeh Saberi Hosnijeh, Barbara Bodinier, Lützen Portengen, Benoît Liquet, Javiera Garrido‐Manriquez, Henk Lokhorst, Ingvar A. Bergdahl, Soterios A. Kyrtopoulos, Ann‐Sofie Johansson, Panagiotis Georgiadis, Beatrice Melin, Domenico Palli, Vittorio Krogh, Salvatore Panico, Carlotta Sacerdote, Rosario Tumino, Paolo Vineis, Raphaële Castagné, Marc Chadeau‐Hyam, on behalf of the EnviroGenoMarkers Consortium Consortium members, Maria Botsivali, Aristotelis Chatziioannou, Ioannis Valavanis, Jos C.S. Kleinjans, Theo M.C.M. de Kok, Hector C. Keun, Toby J. Athersuch, Rachel Kelly, Per Lenner, Goran Hallmans, Euripides G. Stephanou, Antonis Myridakis, Manolis Kogevinas, Lucia Fazzo, Marco De Santis, Pietro Comba, Benedetta Bendinelli, Hannu Kiviranta, Panu Rantakokko, Riikka Airaksinen, Paivi Ruokojarvi, Mark Gilthorpe, Sarah Fleming, Thomas Fleming, Yu‐Kang Tu, Thomas Lundh, Kuo‐Liong Chien, Wei J. Chen, Wen‐Chung Lee, Chuhsing Kate Hsiao, Po‐Hsiu Kuo, Hung Hung, Shu‐Fen Liao

**Affiliations:** ^1^ Division of Environmental Epidemiology Utrecht University, Institute for Risk Assessment Sciences Utrecht The Netherlands; ^2^ MRC‐PHE Centre for Environment and Health, Department of Epidemiology and Biostatistics Imperial College London London United Kingdom; ^3^ Immunology Department Erasmus University Medical Center Rotterdam The Netherlands; ^4^ Laboratoire de Mathématiques et de leurs Applications Université de Pau et des Pays de l'Adour, UMR CNRS Pau France; ^5^ ARC Centre of Excellence for Mathematical and Statistical Frontiers Queensland University of Technology (QUT) Brisbane Australia; ^6^ Department of Hematology University Medical Center Utrecht Utrecht The Netherlands; ^7^ Department of Public Health and Clinical Medicine, and Department of Biobank Research Umeå University Umeå Sweden; ^8^ National Hellenic Research Foundation Institute of Biology, Medicinal Chemistry and Biotechnology Athens Greece; ^9^ Department of Radiation Sciences Oncology, Umeå University Umeå Sweden; ^10^ The Institute for Cancer Research and Prevention Florence Italy; ^11^ Fondazione IRCCS–Instituto Nazionale dei Tumori Milan Italy; ^12^ Department of Clinical Medicine and Surgery University of Naples Frederico II Naples Italy; ^13^ Piedmont Reference Centre for Epidemiology and Cancer Prevention (CPO Piemonte) Turin Italy; ^14^ Cancer registry and Histopathology Unit Azienda Ospedaliera ‘Civile–M.P.Arezzo’ Ragusa Italy; ^15^ HuGeF Foundation Torino Italy; ^16^ INSERM, UMR1027, Université Toulouse III‐Paul Sabatier Toulouse France; ^17^ Institute of Biology, Medicinal Chemistry and Biotechnology National Hellenic Research Foundation Athens Greece; ^18^ Department of Toxicogenomics Maastricht University Maastricht The Netherlands; ^19^ Division of Cancer, Department of Surgery and Cancer Imperial College London, Institute of Reproductive and Developmental Biology (IRDB), Hammersmith Hospital London United Kingdom; ^20^ Division of Computational and Systems Medicine, Department of Surgery and Cancer, Faculty of Medicine Imperial College London London United Kingdom; ^21^ Channing Division of Network Medicine, Department of Medicine Brigham and Women's Hospital and Harvard Medical School Boston USA; ^22^ Nutrition Research, Department of Public Health and Clinical Medicine, and Department of Biobank Research Umeå University Umeå Sweden; ^23^ Environmental Chemical Processes Laboratory University of Crete Heraklion Greece; ^24^ ISGlobal Centre for Research in Environmental Epidemiology (CREAL) Barcelona Spain; ^25^ Istituto Superiore di Sanita Rome Italy; ^26^ National Institute for Health and Welfare Kuopio Finland; ^27^ University of Leeds Leeds United Kingdom; ^28^ Lund University Lund Sweden; ^29^ National Taiwan University Taipei Taiwan

**Keywords:** lymphoma, multiple myeloma, cytokine, prospective cohort, mixed‐effect modeling, multivariate models, time to diagnosis

## Abstract

Recent prospective studies have shown that dysregulation of the immune system may precede the development of B‐cell lymphomas (BCL) in immunocompetent individuals. However, to date, the studies were restricted to a few immune markers, which were considered separately. Using a nested case–control study within two European prospective cohorts, we measured plasma levels of 28 immune markers in samples collected a median of 6 years before diagnosis (range 2.01–15.97) in 268 incident cases of BCL (including multiple myeloma [MM]) and matched controls. Linear mixed models and partial least square analyses were used to analyze the association between levels of immune marker and the incidence of BCL and its main histological subtypes and to investigate potential biomarkers predictive of the time to diagnosis. Linear mixed model analyses identified associations linking lower levels of fibroblast growth factor‐2 (FGF‐2 *p* = 7.2 × 10^−4^) and transforming growth factor alpha (TGF‐α, *p* = 6.5 × 10^−5^) and BCL incidence. Analyses stratified by histological subtypes identified inverse associations for MM subtype including FGF‐2 (*p* = 7.8 × 10^−7^), TGF‐α (*p* = 4.08 × 10^−5^), fractalkine (*p* = 1.12 × 10^−3^), monocyte chemotactic protein‐3 (*p* = 1.36 × 10^−4^), macrophage inflammatory protein 1‐alpha (*p* = 4.6 × 10^−4^) and vascular endothelial growth factor (*p* = 4.23 × 10^−5^). Our results also provided marginal support for already reported associations between chemokines and diffuse large BCL (DLBCL) and cytokines and chronic lymphocytic leukemia (CLL). Case‐only analyses showed that Granulocyte‐macrophage colony stimulating factor levels were consistently higher closer to diagnosis, which provides further evidence of its role in tumor progression. In conclusion, our study suggests a role of growth‐factors in the incidence of MM and of chemokine and cytokine regulation in DLBCL and CLL.

## Abstract 2

**What's new?**

B‐cell lymphomas (BCL) are frequent in immunocompromised individuals, but most BCL cases are thought to occur as a consequence of minor immune perturbations in otherwise immunocompetent individuals. Here the authors prospectively examined a panel of immune markers in the blood from 268 patients afflicted with BCL and paired controls. The data uncover a functional role for growth factors (i.e. FGF‐2, TGF‐alpha) in the incidence and progression of multiple myeloma, a BCL subtype, and underscore the importance of chemokine and cytokine regulation in diffuse large B‐cell lymphoma and chronic lymphocytic leukemia.

## Introduction

B‐cell lymphomas (BCLs) are the most common hematopoietic cancers in both men and women in the developed world.^1^, ^2^ The strongest and most consistent risk factors are related to altered immunity conditions including HIV infection or iatrogenically induced immune suppression after transplantation.^1^ However, the prevalence of these conditions is too low to explain the majority of BCL cases. This has led to the hypothesis that minor perturbations in immune function among otherwise immunocompetent individuals could be related to future BCL risk.

Few prospective studies have reported on the possible association linking BCL risk and circulating levels of immune markers. These studies have suggested an increased risk of BCL and or its subtypes with increased blood levels of soluble CD (sCD)23,^3^ sCD27,^3^, ^4^, ^5^ sCD30,^3^, ^4^, ^5^, ^6^, ^7^ soluble interleukin (sIL)‐2R,^8^ tumor necrosis factor (TNF),^9^ sTNF‐R1,^10^ sTNF‐R2,^10^, ^11^ BCA‐1,^5^, ^12^ TNF‐α,^10^, ^12^ soluble vascular endothelial growth factor receptor (sVEGFR)2,^11^ sIL‐6R^13^ and IL10,^9^, ^10^, ^14^ while IL13^8^ was negatively associated with the risk of BCL. Most of these studies investigated the associations looking at proteins separately. However, as many immune markers are pleiotropic and exert multiple (overlapping) effects, methods modeling possible joined effects of these molecules may be more appropriate to investigate future risk of BCL.

In our study, we use blood samples collected years before clinical diagnosis to interrogate the relationship between pre‐diagnostic blood levels of a large panel of cytokines, chemokines and growth factors and future risk of BCL and its main histological subtypes. We investigate the marginal relationship of each inflammatory biomarkers separately through univariate analyses, and consider the potential for a joint effect of these markers through penalized multivariate models (in our case, partial least square [PLS]). Exploiting the prospective nature of our biosamples, we also seek for (combinations of) biomarkers that would be indicative of the time elapsed between protein measurement and clinical onset.

## Material and Methods

### Study subjects

The EnviroGenoMarkers (EGM) study^15^ is based on participants from two existing prospective cohorts: the Italian component of the European Prospective Investigation into Cancer and Nutrition (EPIC‐Italy)^16^ and the Northern Sweden Health and Disease Study (NSHDS).^17^ In both cohorts, blood samples were (prospectively) collected from healthy subjects at enrolment. This study was approved by the committee on research ethics at the relevant institutions.

The EPIC project is a European network of prospective cohorts that was set up to examine relationships of cancer risk with nutrition and metabolic risk factors.^18^ Between 1993 and 1998, EPIC Italy completed the recruitment of 47,749 volunteers (15,171 men and 32,578 women, ages 35–70 years) in five different areas covered by cancer registries.^16^ After providing informed consent, a blood sample was collected as well as detailed information on dietary and life‐style habits using standardized questionnaires (http://epic.iarc.fr/research/quest.php). Incident primary cancer was identified by automated linkages to cancer and mortality registries, population offices of all municipalities where participants reported to be residing, hospital discharge systems and periodic personal contacts (in Naples).

The NSHDS cohort contains three sub‐cohorts, of which we solely used samples from the Västerbotten Intervention Program. A total of 80,000 healthy individuals aged 40–60 years were recruited between 1985 and 2008 and were asked to complete a self‐administered questionnaire collecting demographic, medical and lifestyle information as well as a separate self‐administered food frequency questionnaire. Informed consent was obtained from all participants and a medical examination was conducted during which a blood sample was taken. Incidence cancers occurring among cohort members during the study period were identified by linkage with the Swedish Cancer Registry and the local Northern Sweden Cancer Registry.

For both cohorts, within 2 hours of blood collection, blood samples were processed for the isolation of buffy coats and other fractions which were placed in cold storage (liquid N_2_ in EPIC‐Italy and −80°C in NSHDS). Samples were transported on dry ice to the laboratory and stored for a short period at −80°C before analyses.

For each incident BCL case identified within the two cohorts, one random control was selected among all cohort members alive and free of cancer at the time of diagnosis of the index case matched by cohort, center, gender, date of blood collection (± 6 months) and age at recruitment (± 2.5 years). Information from the two studies was integrated into a single database and standardized. Lymphoma cases were classified into subtypes according to the SEER ICD‐O‐3 morphology codes.^19^ We considered multiple myeloma (MM) together with all other BCL subtypes and hereafter in the text BCL includes MM.

We first analyzed samples from 100 case–control pairs (phase 1), which were subsequently supplemented with an additional 181 case–control pairs to increase the power of the study (147 cases in NSHDS, 34 cases in EPIC‐Italy) (phase 2).^19^ After further subtype characterization and review, 11 cases were reclassified (Hodgkin's lymphoma [*n* = 6]; T‐cell lymphoma [*n* = 1]; and unknown (*n* = 4)) and excluded from further analysis along with their matched controls. Moreover, two cases without suitable control samples were excluded. The final number of included successfully analyzed samples was 268 BCL cases and 268 controls (Table 1). Median time between blood collection and diagnosis of BCL was 6.2 years (range, 2.01–15.97) in NSHDS and 5.4 (range, 2.03–11.47) in EPIC‐Italy.

**Table 1:** General characteristics of BCL cases and matched controls

Baseline variable	Cases (*n* = 268)	Controls (*n* = 268)	*p* value^a^
Cohort, *n* (%)			
EPIC‐Italy	84 (31.3)	84 (31.3)	
NSHDS	184 (68.7)	184 (68.7)	
Phase, *n* (%)			
1	96 (35.8)	96 (35.8)	
2	172 (64.2)	172 (64.2)	
Sex, *n* (%)			
Male	132 (49.3)	132 (49.3)	
Female	136 (50.7)	136 (50.7)	
Age at recruitment (years)^2^	53.1 (7.8)	53.1 (7.8)	0.99
Alcohol intake (g/day)^2^	7.05 (12.5)	8.25 (14.7)	0.32
Body mass index (kg/m^2^)^2^	26.36 (3.8)	26.53 (4.1)	0.57
Smoking Status, *n* (%)			0.80
Never	145 (54.1)	150 (56.0)	
Former	68 (25.4)	63 (23.5)	
Current	55 (20.5)	55 (20.5)	
Highest educational level, *n* (%)			0.28
None	4 (1.5)	1 (0.4)	
Primary	96 (35.8)	104 (38.8)	
Technical/professional	68 (25.4)	56 (20.9)	
Secondary	53 (19.8)	65 (24.3)	
University/college	47 (17.5)	42 (15.7)	
Physical activity (Cambridge index), *n* (%)			0.22
Inactive	80 (29.9)	76 (28.4)	
Moderately inactive	106 (39.6)	95 (35.4)	
Moderately active	68 (25.4)	74 (27.6)	
Active	14 (5.2)	23 (8.6)	
BCL sub‐types, *n* (%)^3^			
DLBCL	44 (16.4)		
FL	39 (14.6)		
CLL	42 (15.6)		
MM	76 (28.4)		
Other subtypes	67 (25)		

Abbreviations: CLL, chronic lymphocytic leukemia; DLBCL, diffuse large BCL; FL, follicular lymphoma; MM, multiple myeloma; EPIC, European Prospective Investigation into Cancer and Nutrition; and NSHDS, the Northern Sweden Health and Disease Study.

a *P* values are based on t‐tests for continuous variables and χ^2^ tests for categorical variables. ^2^Mean (standard deviation). ^3^Number of cases for each subtype in EPIC‐Italy: DLBCL = 11, CLL = 11, FL = 20, MM = 21, others = 21, and in NSHDS: DLBCL = 33, CLL = 31, FL = 19, MM = 55, others = 46.

For 224 case–control pairs (from both cohorts), full‐resolution DNA methylation data were also available from Illumina Infinium Human Methylation 450 platform using standard protocol and preprocessing/normalizing steps as described elsewhere.^20^ From these profiles, using an established deconvolution approach,^21^ we estimated the proportion of the following six blood components: CD4, CD8 and natural T‐cells, B‐cells, monocytes and granulocytes.

### Measurement of immune markers

Blood samples were collected in citrate (Italy) or ethylene diamine tetraacetic acid (EDTA) (Sweden) tubes and processed by centrifugation within 2 hrs after collection. We measured a large panel of inflammation‐related proteins (*n* = 32) including IL1β, IL2, IL4, IL5, IL6, IL7, IL8, IL10, IL12, IL13, interferon alpha (INF‐α), INF‐γ, TNF‐α, eotaxin, IL1 receptor antagonist (IL1‐RA), sIL‐2RA, INF‐γ‐induced protein 10 (IP10), granulocyte–macrophage colony stimulating factor (GMSCF), epidermal growth factor (EGF), fibroblast growth factor 2 (FGF‐2), fms‐like tyrosine kinase receptor‐3 (Flt3) ligand protein (Flt3ligand), fractalkine, granulocyte colony‐stimulating factor (GCSF), melanoma growth stimulatory activity/growth‐related oncogene (GRO), monocyte chemotactic protein‐1 (MCP‐1), MCP‐3, macrophage‐derived chemokine (MDC), macrophage inflammatory protein 1 alpha (MIP‐1α), macrophage inflammatory protein 1 beta (MIP‐1ß), soluble CD40 ligand (sCD40L), transforming growth factor alpha (TGF‐α) and VEGF in stored plasma samples of all cases and controls using the milliplex HCYTOMAG‐60K and HSCYTMAG‐60SK kits (Millipore, Billerca, MA), according to the protocol described by the manufacturer. All measured markers have previously been investigated at least in one study, except fractalkine, MCP‐3 and INF‐α.

Laboratory personnel were blinded with regard to case–control status. Cases and controls were assayed next to each other on the same plate in the same batch and a single quality control sample was run in duplicate with the case–control sets in each plate. Samples of phase 1 were run once due to sample volume limitations while samples in phase 2 were run in duplicate. Four analytes (IL12, IL1‐RA, sIL2‐RA and Flt3ligand) were excluded from further statistical analyses due to a high rate of non‐detects (>75%). Median intra‐batch coefficients of variation (CV) for all cytokines based on the quality control duplicates was 14.8 and 5.7 and median inter‐batch CV was 7.7 and 13.3 for phase 1 and 2, respectively (Supporting Information Table S1). Median intra class correlation coefficient (ICC) of the measured analytes was 0.87 and was above 0.5, except for MDC and FGF‐2 (0.16 and 0.43, respectively) (Supporting Information Table S1). Cytokine levels measured out of range of the calibration curve (either too low: <limit of detection (LOD), or too high) and missing values for covariate (body mass index [*n* = 8], smoking status [*n* = 14], education [*n* = 16], alcohol intake [*n* = 41], physical activity [*n* = 2]) were imputed based on a maximum likelihood estimation method which was informed by the observed correlation structure within the data.^22^ Imputation of samples <LOD was carried out using the empirical LOD across all plates as the upper bound. For imputation of samples with a concentration exceeding the calibration curve, we used a value of twice the highest observed concentration that was not out of range as the upper bound. 70% of the retained markers had <30% imputed values (Supporting Information Table S1). In all analyses, levels of cytokines were log‐transformed to normalize their distributions. Differences between cases and controls in baseline continuous covariates were assessed using paired Student's *t test*, and a χ^2^ test for categorical variables.

### Linear mixed models

As proposed elsewhere,^19^ linear mixed models were used to investigate the relationship between each of the immune marker levels separately and the disease outcome. The general formulation of the mixed model for a given protein (continuous variable) observed in participant *i (Y^i^)* can be described as follows:

$$Y^{i}∼\alpha +\beta_{1}X^{i}+\beta_{2}FE^{i}+u^{Ai}+{ɛ}^{i},$$

where *α* is the intercept, *ɛ^i^* is the residual error and *X^i^* is a binary variable indicating whether individual *i* is a BCL case or not. *FE* ^i^ is a vector of fixed effect observations for individual *I,* including the matching criteria (age, gender and country), the experimental phase (1 or 2) and as potential confounders, body mass index (BMI, continuous, in kg/m2), education (categorical: none, primary, technical/professional, secondary, university/college), physical activity (categorical: inactive, moderately inactive, moderately active, active), smoking status at enrolment (categorical: non‐smokers, former smokers, smokers) and alcohol intake at enrolment (continuous in g/day). Nuisance variation due to differences between microtiter plates was modeled through a random intercept *u^Ai^* (where *Ai* denotes the plate on which sample *i* was assayed).

The strength of the association between the BCLs (or histological subtypes) case/control status and each protein level was inferred using a likelihood ratio test comparing the model with the disease status (*X^i^*) variable to the one without it. The model was fitted on all markers separately, and we accounted for multiple testing using a Bonferroni correction, controlling the family wise error rate (FWER) below 5%.

To investigate potential confounding by blood cell count differentials, the models (for full BCL and all histological subtypes) were further adjusted on the estimated cell proportions for 5 (of the 6) cell types (CD8, CD4, natural killer T cells, B cells and monocytes) for the 224 pairs in which that information was available.

Proteins that were found to be differentially expressed between cases and controls were further investigated through unconditional logistic regression (ULR), where, for a given subtype, cases were compared to all controls, adjusting for country, gender, age at recruitment, phase and microtiter plate number. Quartiles (Q) of plasma cytokine concentrations were calculated based on the distribution in controls.

### PLS analyses

To evaluate the potential for a joint inflammatory signal that would be related to BCL or any of its histological subtypes, we performed series of PLS‐DA analyses in relation to case/control status. To facilitate interpretability, we performed variable selection by penalizing the loadings coefficients as proposed in the sparse PLS‐DA models (sPLS‐DA).^23^ As recently proposed,^24^ we also accounted for a functional grouping of the proteins in cytokines, chemokines and growth factors classes to inform the model. We ran series of sparse group PLS‐DA (sgPLS‐DA) analyses to select the most relevant protein groups in relation to disease status and imposed sparsity within the selected groups. In all PLS‐DA analyses, the number of components was set to 1, and calibration of both the penalty, and, when applicable (sGPLS‐DA), of the number of selected groups was done *via* fivefold cross‐validation repeated 100 times. Calibration parameters were chosen to minimize the average misclassification rates, using univariate and bi‐dimensional grids for sPLS‐DA and sgPLS‐DA, respectively, exploring all possible values of the number of selected variables and, if applicable, groups.

We also adopted an sPLS approach to investigate the relationship between prediagnostic levels of inflammatory markers in BCL cases and the time elapsed between the measurement and the clinical onset. We ran these analyses for BCL cases only and calibrated the penalty using the same cross‐validation procedure to minimize, in that case, the mean square error of prediction.

For all PLS‐DA and PLS analyses, we conducted a series of stability analyses randomly sub‐sampling (*N* = 10,000 times) 80% of the study population, and running the PLS models for each subsample. As a measure of stability, we report, for all investigated values of the calibration parameters, the number of times each variable was selected across the 10,000 subsamples.

To adjust results from all PLS analyses for technically induced variation, and as already proposed,^25^, ^26^ we inferred de‐noised data from the linear mixed model presented above by subtracting from the observations the estimated random effects.

Linear mixed models were fitted using lme4 R‐statistical package, and all PLS and PLS‐DA analyses were performed using the R‐statistical package sgPLS using the R 3.4.0 language and environment (The R Foundation for Statistical Computing, Vienna, Austria). Conditional and ULRs were performed using SAS (ver. 9.2, SAS institute).

## Results

Of all BCL cases, 16.4% were diagnosed with diffuse large BCL (DLBCL) (*n* = 44), 14.6% with follicular lymphoma (FL) (*n* = 39), 15.6% with CLL (*n* = 42) and 28.4% with MM (*n* = 76). Distribution of BCL subtypes and gender across phases and countries are shown in Supporting Information Table S2. Each phase includes subjects from both cohorts and gender. Characteristics of the study population are summarized in Table 1. Supporting Information Tables S3 and S4 show the median, minimum and maximum levels of all cytokines stratified by case–control status, country, phase of study and BCL subtypes. Median concentration of most immune markers was higher among control subjects, phase 1 and NSHDS subjects compared with cases, phase 2 and EPIC‐Italy subjects, respectively.

### Linear mixed model analyses

**Figure 1:**
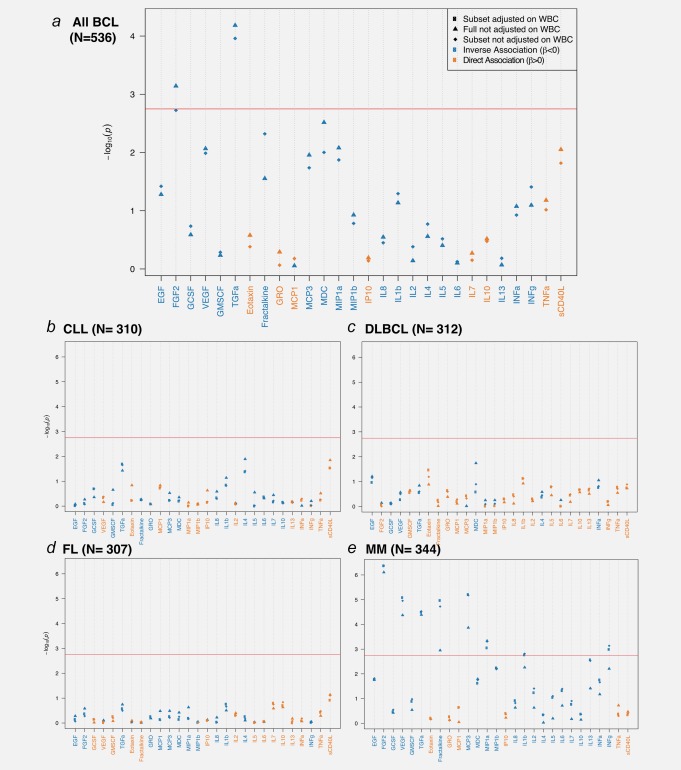
Results of the mixed model analyses between log‐transformed values of immune markers and all BCL case–control status (*a*, *N* = 268 pairs). Results are also presented for main histological subtypes: CLL (*b*, *N* = 42 cases and 268 controls), DLBCL (*c*, *N* = 44 cases and 268 controls), FL (*d*, *N* = 39 cases and 268 controls) and MM (*e*, *N* = 76 cases and 268 controls). Strength of association (Y‐axis) is measured by *p* values and the black horizontal line represents the Bonferroni cut‐off value ensuring a control of the FWER below 0.05. Inverse associations are represented in blue and positive associations in orange, and the names of the proteins are colored accordingly. Results are presented for the full study population, and for the (*N* = 224) pairs in which WBC composition estimates were available (*N* = 224 case/control pairs), models are either adjusted (squares) or unadjusted (diamonds) of WBC proportions. Abbreviations: IL, interleukin; INF‐α, interferon alpha; INF‐γ, interferon gamma; GMCSF, granulocyte–macrophage colony stimulating factor; TNF‐α, tumor necrosis factor alpha; EGF, epidermal growth factor; FGF‐2, fibroblast growth factor 2; GCSF, granulocyte colony‐stimulating factor; GRO, melanoma growth stimulatory activity/growth‐related oncogene; IP10, INF‐γ‐induced protein 10; MCP‐1, monocyte chemotactic protein‐1; MCP‐3, monocyte chemotactic protein‐3; MDC, macrophage derived chemokine; MIP‐1α, macrophage inflammatory protein 1 alpha; MIP‐1ß, macrophage inflammatory protein 1 beta; sCD40L, soluble CD40 ligand; VEGF, vascular endothelial growth factor; TGF‐α, transforming growth factor alpha. [Color figure can be viewed at http://wileyonlinelibrary.com]

In a first set of analyses, all BCL cases were pooled together and multivariate analyses revealed a general lower level of inflammatory markers among cases compared with controls (Fig. 1 *a*, Supporting Information Tables S3 and S5). Among the 28 analytes, 20 showed an inverse association with disease status. Of these, only two reached Bonferroni significance level (Supporting Information Table S5) and involved blood levels of FGF‐2 (*β* = –0.50, *p* values = 7.2 × 10^−4^) and TGF‐α (*β* = –0.68, *p* values = 6.5 × 10^−5^). Models adjusted for white blood cell (WBC) differentials provided consistent results (Supporting Information Table S6) and one borderline significant association involving fractalkine (*β* = –0.47, *p* values = 1.84 × 10^−3^) emerged.

Stratification upon histological subtype did not show any significant associations (FWER < 5%) with CLL, DLBCL and FL (Figs. 1 *b*–1 *d*, respectively, and Supporting Information Table S5). In contrast, we identified six inverse associations for MM subtype (Fig. 1 *e*). We found that lower plasma levels of FGF‐2 (*β* = –1.10, *p* values = 7.8 × 10^−7^), fractalkine (*β* = –0.72, *p* values = 1.12 × 10^−3^), MCP‐3 (*β* = –0.91, *p* values = 1.36 × 10^−4^), MIP‐1α (*β* = –0.72, *p* values = 4.6 × 10^−4^), TGF‐α (*β* = –1.08, *p* values = 4.08 × 10^−05^), VEGF (*β* = –1.00, *p* values = 4.23 × 10^−5^) were associated with increased risk of MM (Supporting Information Table S5). Upon adjustment for WBC proportion, two additional associations involving INF‐γ (*β* = –0.96, *p* values = 1.03 × 10^−3^) and IL1β (*β* = –0.71, *p* values = 1.71 × 10^−3^) emerged (Supporting Information Table S6). Stratified analyses by phase showed that the results for MM were consistent between the analyses done in Phases 1 and 2, providing technical replication of our results (Supporting Information Tables S7*a* and S7*b*). Given that the two associations found in the pooled BCL analyses correspond to the two strongest MM‐specific associations, one can hypothesize that these findings are driven by the MM subtype. This was confirmed by additional analyses of pooled BCL excluding MM cases, where no significant associations were observed (Supporting Information Fig. 1).

Linear mixed models were further stratified by median time‐to‐diagnosis: before 6 years (Supporting Information Table S8) and after 6 years (Supporting Information Table S9) for all BCL, BCLL, MM and DLBCL subtypes. Results showed largely similar associations for cases diagnosed within or >6 years after blood collection.

Results from the multivariable ULR models (including all lymphoma controls) for MM were consistent with the linear regression analyses. These identified an inverse association between risk of MM (Table 2) and blood levels of FGF‐2 (OR = 0.22, for 4^th^Q *vs*. 1^st^Q, P‐ trend < 0.0001), fractalkine (OR = 0.25, for 4^th^Q *vs*. 1^st^Q, P‐trend = 0.003), MCP‐3 (OR = 0.34, for 4^th^Q *vs*. 1^st^Q, P‐trend = 0.003), MIP‐1α (OR = 0.38, for 4^th^Q *vs*. 1^st^Q, P‐trend = 0.02), TGF‐α (OR = 0.33, for 4^th^Q *vs*. 1^st^Q, P‐trend = 0.0003) and VEGF (OR = 0.35, for 4^th^Q *vs*. 1^st^Q, P‐trend = 0.001). Results were consistent in both cohorts although strength of the associations was reduced, especially for EPIC‐Italy, possibly owing to the lower number of cases. Analyses comparing MM cases to individually age‐ and sex‐matched controls essentially rendered similar results (Supporting Information Table S10). Moreover, analyses including minimal adjustment (excluding some factors which are weak confounders such as smoking status and physical activity) did not affect the presented results.

**Table 2:** Risk of MM, stratified by cohort, by quartiles of circulating immune markers

Immune markers		All cases		EPIC‐Italy		NSHDS	
		Ca/Co, *n*	OR (95% CI)	*n*	OR (95% CI)	*n*	OR (95% CI)
FGF‐2	Q1 (<1.67)	34/67	Ref.	9/18	Ref.	25/49	Ref.
	Q2 (1.67–3.23)	24/67	0.72 (0.42–1.22)	6/19	0.48 (0.15–1.56)	18/48	0.69 (0.37–1.29)
	Q3 (3.24–4.32)	12/67	0.41 (0.21–0.82)	4/24	0.43 (0.12–1.59)	8/43	0.38 (0.17–0.86)
	Q4 (>4.32)	6/67	0.22 (0.09–0.53)	2/23	0.17 (0.03–0.85)	4/44	0.20 (0.07–0.61)
	*P*‐trend		<0.0001		0.02		0.001
Fractalkine	Q1 (<2.51)	31/67	Ref.	5/7	Ref.	26/60	Ref.
	Q2 (2.51–3.91)	19/67	0.72 (0.40–1.30)	5/28	0.53 (0.13–2.14)	14/39	0.81 (0.42–1.58)
	Q3 (3.92–5.07)	20/67	0.68 (0.37–1.23)	10/22	1.16 (0.32–4.26)	10/45	0.51 (0.24–1.10)
	Q4 (>5.07)	6/67	0.25 (0.10–0.62)	1/27	0.12 (0.01–1.13)	5/40	0.32 (0.12–0.87)
	*P*‐trend		0.003		0.19		0.01
MCP‐3	Q1 (<0.62)	33/67	Ref.	7/14	Ref.	26/53	Ref.
	Q2 (0.62–2.39)	18/67	0.66 (0.37–1.20)	7/28	0.61 (0.18–2.04)	11/39	0.62 (0.30–1.26)
	Q3 (2.40–3.50)	16/67	0.57 (0.31–1.05)	6/21	0.66 (0.19–2.26)	10/46	0.52 (0.25–1.09)
	Q4 (>3.50)	9/67	0.34 (0.16–0.72)	1/21	0.11 (0.01–0.95)	8/46	0.39 (0.17–0.90)
	*P*‐trend		0.003		0.05		0.02
MIP‐1α	Q1 (<0.59)	22/67	Ref.	7/14	Ref.	15/53	Ref.
	Q2 (0.59–2.43)	29/67	1.22 (0.69–2.15)	7/17	1.34 (0.36–5.03)	22/50	1.33 (0.69–2.59)
	Q3 (2.44–3.49)	17/67	0.74 (0.39–1.42)	5/17	0.94 (0.26–3.35)	12/50	0.79 (0.36–1.73)
	Q4 (>3.49)	8/67	0.38 (0.16–0.91)	2/36	0.21 (0.04–1.10)	6/31	0.50 (0.17–1.45)
	*P*‐trend		0.02		0.04		0.17
TGF‐α	Q1 (<–0.55)	33/67	Ref.	8/16	Ref.	25/51	Ref.
	Q2 (–0.55–0.77)	26/67	0.82 (0.48–1.39)	9/21	1.02 (0.34–3.01)	17/46	0.71 (0.38–1.35)
	Q3 (0.78–2.38)	8/67	0.31 (0.14–0.68)	2/19	0.31 (0.06–1.49)	6/48	0.29 (0.12–0.73)
	Q4 (>2.38)	9/67	0.33 (0.15–0.70)	2/28	0.19 (0.04–0.97)	7/39	0.35 (0.14–0.87)
	*P*‐trend		0.0003		0.01		0.003
VEGF	Q1 (<3.31)	30/67	Ref.	11/17	Ref.	19/50	Ref.
	Q2 (3.31–5.28)	27/67	0.91 (0.54–1.55)	6/20	0.66 (0.23–1.89)	21/47	1.05 (0.55–2.00)
	Q3 (5.29–6.52)	10/67	0.38 (0.18–0.78)	2/21	0.24 (0.05–1.17)	8/46	0.45 (0.19–1.07)
	Q4 (>6.52)	9/67	0.35 (0.16–0.75)	2/26	0.18 (0.04–0.87)	7/41	0.40 (0.16–1.03)
	*P*‐trend		0.001		0.01		0.02

Quartiles of (log‐transformed) plasma levels of immune markers were calculated based on the distribution in control subjects. Models adjusted for age, sex, country, body mass index, smoking status, education, physical activity, alcohol intake and phase. Tests for trend were calculated using the quartile number as continuous variable. Noise variance due to plate was removed before analyses.

Abbreviations: FGF‐2, fibroblast growth factor‐2; TGF‐α, transforming growth factor alpha; MCP‐3, monocyte chemotactic protein‐3; MIP‐1α, macrophage inflammatory protein‐1 alpha; VEGF, vascular endothelial growth factor; EPIC, European Prospective Investigation into Cancer and Nutrition; NSHDS, the Northern Sweden Health and Disease Study.

### Multivariate analyses: PLS‐DA models

**Figure 2:**
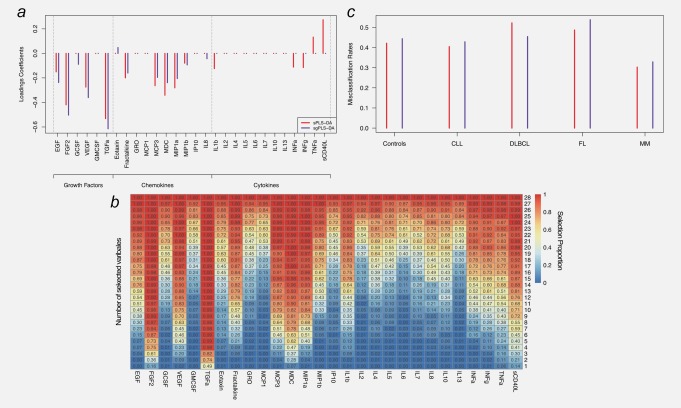
Results of the sparse and sparse group PLS‐DA (sPLS‐DA and sgPLS‐DA, respectively) analyses for all BCL cases. Loadings coefficients are presented for sPLS‐DA and sgPLS‐DA (*a*). Results from the stability analyses of sPLS‐DA analyses subsampling (*N* = 10,000 times) 80% of the study population are summarized in (*b*) and represents for each protein (X‐axis) and each possible number of selected variables (Y‐axis), the selection proportion (across the 10,000 repeats). Misclassification rates for each variant of the PLS algorithm used are presented in (*c*), for controls and each BCL subtype separately. Abbreviations: IL, interleukin; INF‐α, interferon alpha; INF‐γ, interferon gamma; GMCSF, granulocyte–macrophage colony stimulating factor; TNF‐α, tumor necrosis factor alpha; EGF, epidermal growth factor; FGF‐2, fibroblast growth factor 2; GCSF, granulocyte colony‐stimulating factor; GRO, melanoma growth stimulatory activity/growth‐related oncogene; IP10, INF‐γ‐induced protein 10; MCP‐1, monocyte chemotactic protein‐1; MCP‐3, monocyte chemotactic protein‐3; MDC, macrophage derived chemokine; MIP‐1α, macrophage inflammatory protein 1 alpha; MIP‐1ß, macrophage inflammatory protein 1 beta; sCD40L, soluble CD40 ligand; VEGF, vascular endothelial growth factor; TGF‐α, transforming growth factor alpha. [Color figure can be viewed at http://wileyonlinelibrary.com]

Calibration of the sPLS‐DA analysis of all BCL cases selected 14 variables as yielding optimal balance between discriminatory performances and sparsity. Of these, 12 showed negative loadings coefficients, suggesting consistent lower levels of inflammatory markers in BCL cases (Fig. 2 *a*). Stability analyses for models of size 14 showed that 8 of the 14 proteins were selected in >80% of the (*N* = 10,000) subpopulations. In particular, extended stability analyses to all possible number of variable selected in the sPLS‐DA component (Fig. 2 *b*) showed that both FGF‐2 and TFGα were the first variables to be consistently selected, even in sparse models: both proteins showed selection proportions above 70% in (sub‐optimal) models selecting five variables.

Calibration of the sparse group PLS (sgPLS‐DA, Fig. 2 *a*) selected two groups (growth factors and chemokines) and selected five proteins with negative loadings coefficients in the growth factors group (including EGF, FGF‐2, GCSF, VEGF and TGF‐α), and seven proteins in the chemokines group: fractalkine, MCP‐3, MDC, MIP‐1a, MIP‐1b, IL8 with negative loadings and eotaxin, with positive (although lower in absolute value) loadings coefficients. We further explored different functional groupings based on predominant origin or affinity of the measured cytokines (myeloid, lymphoid, other; B‐cell, T‐cell, B&T‐cell, other; macrophages, granulocytes, eosinophils *etc*.). These analyses did not change the results presented here (not shown).

**Figure 3:**
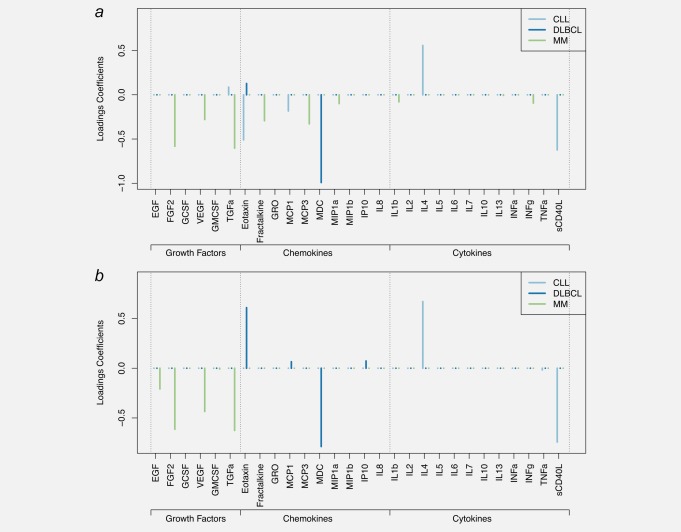
Results of the sparse PLS‐DA (sPLS‐DA) and sparse group PLS‐DA (sgPLS‐DA). Models were fitted on subtype‐specific sets of cases and controls. Loadings coefficients are presented for DLBCL, CLL and MM separately for the sPLS‐DA model (*a*), and the sgPLS‐DA (*b*) models. Models for FL are not reported as they yielded poor predictive performances. Abbreviations: IL, interleukin; INF‐α, interferon alpha; INF‐γ, interferon gamma; GMCSF, granulocyte–macrophage colony stimulating factor; TNF‐α, tumor necrosis factor alpha; EGF, epidermal growth factor; FGF‐2, fibroblast growth factor 2; GCSF, granulocyte colony‐stimulating factor; GRO, melanoma growth stimulatory activity/growth‐related oncogene; IP10, INF‐γ‐induced protein 10; MCP‐1, monocyte chemotactic protein‐1; MCP‐3, monocyte chemotactic protein‐3; MDC, macrophage derived chemokine; MIP‐1α, macrophage inflammatory protein 1 alpha; MIP‐1ß, macrophage Inflammatory Protein 1 beta; sCD40L, soluble CD40 ligand; VEGF, vascular endothelial growth factor; TGF‐α, transforming growth factor alpha. [Color figure can be viewed at http://wileyonlinelibrary.com]

Misclassification rates yielded by the calibrated sPLS‐DA and sgPLS‐DA analyses for all BCL cases (Fig. 3 *c*) showed rather high error rates, hence indicating a moderate predictive value of these combinations of markers (AUC from ROC analyses were below 62% for both models). However, our results clearly suggest that, irrespective of the variant of the PLS‐DA model, the model fitted on all BCL cases showed lower misclassification rates for the MM cases (Fig. 2 *c*).

Calibration of the subtype‐specific sPLS‐DA and sgPLS‐DA models were restricted to cases control pairs within each subtype (*N* = 42, 44, 39 and 76 pairs for CLL, DLBCL, FL and MM respectively), and models for FL yielded poor discriminatory performances with misclassification rates higher than 58%. Due to this, we do not report FL‐related results.

**Figure 4:**
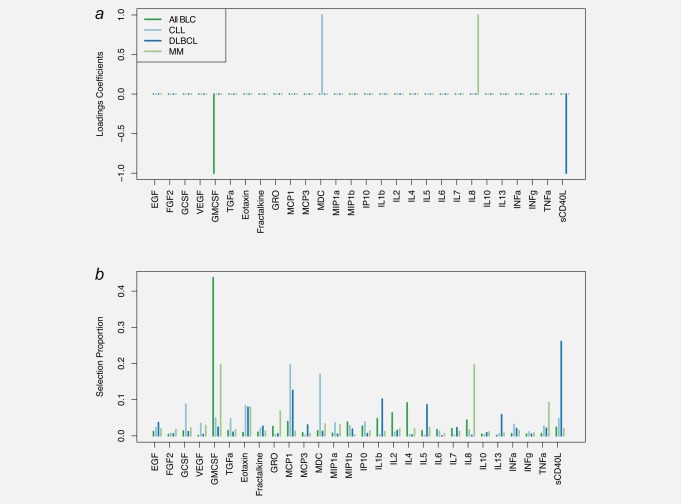
Results of the sparse PLS analyses of time to diagnosis in cases only. Results are presented for all BCL cases and for cases of DLBCL, CLL and MM separately. Loadings coefficients obtained for the calibrated models are presented for each set of cases considered (*a*). Results from stability analyses using 10,000 subsamples of the full set of cases are represented in B by the per‐variable proportion of selection across all independent subsamples. Abbreviations: IL, interleukin; INF‐α, interferon alpha; INF‐γ, interferon gamma; GMCSF, granulocyte–macrophage colony stimulating factor; TNF‐α, tumor necrosis factor alpha; EGF, epidermal growth factor; FGF‐2, fibroblast growth factor 2; GCSF, granulocyte colony‐stimulating factor; GRO, melanoma growth stimulatory activity/growth‐related oncogene; IP10, INF‐γ‐induced protein 10; MCP‐1, monocyte chemotactic protein‐1; MCP‐3, monocyte chemotactic protein‐3; MDC, macrophage derived chemokine; MIP‐1α, macrophage inflammatory protein 1 alpha; MIP‐1ß, macrophage inflammatory protein 1 beta; sCD40L, soluble CD40 ligand; VEGF, vascular endothelial growth factor; TGF‐α, transforming growth factor alpha. [Color figure can be viewed at http://wileyonlinelibrary.com]

As depicted in Figure 3 *a*, sets of selected variables showed limited overlap across histological subtypes. For DLBCL, two variables were selected by the sPLS‐DA (Fig. 4 *a*): MDC (negative loadings) and eotaxin (positive loadings). Including a group structure in the model (Fig. 3 *b*), resulted in only the chemokines being selected to discriminate DLBCL cases and controls, and within chemokines, MDC and eotaxin were selected with highest loadings (in absolute value) along with MCP‐1 and IP10. Stability analyses (Supporting Information Fig. 2*a*) suggested that these two proteins were the only ones stably selected in sparse models.

For CLL, five variables were selected by sPLS‐DA (Fig. 3 *a*): IL4 and TGF‐α (positive loadings coefficient, and sCD40L, eotaxin and MCP‐1 (with negative loadings coefficients). Stability analyses (Supporting Information Fig. 2*b*) showed that IL4, eotaxin and sCD40L showed high selection probability (>70%) for models including five or more variables. Sparse group PLS‐DA models for CLL selected a single group (cytokines) and within the group, sCD40L and IL4 (Fig. 3 *b*).

MM analyses selected eight variables (all negative loadings coefficients, Fig. 3 *a*). Of these, five showed higher absolute values of the loadings coefficients and high selection proportion (>75% selection proportion for models of size 8; Supporting Information Fig. 2*c*): FGF‐2, TGF‐α, MCP‐3, factalkine and VEGF. Calibration of the sgPLS‐DA analyses of MM subtype selected a single group: growth factors (Fig. 3 *b*) and within this group EGF, FGF‐2, VEGF and TGF‐α were selected.

To explicitly model the relationship between our 28 inflammatory markers and the time to diagnosis, we ran series of sPLS analyses in cases only (all BCL cases and subsequently DLBCL, CLL and MM separately), relating prospective blood levels of all inflammatory markers and the observed time to diagnosis. The resulting sPLS models (Fig. 4 *a*) selected a single variable for all BCL (GMCSF, negative loadings coefficient), for DLBCL (sCD40L, negative loadings coefficient), for MM (IL8, positive loadings coefficient) and CLL (MDC, positive loadings coefficient). Stability analyses (Fig. 4 *b*) showed that while each of these subtype‐specific variables were the most frequently selected, especially for all BLC analyses (selection proportion of GMCSF >40%), other variables were competing to predict time to diagnosis. For MM both IL8 and GMCSF were selected in 20% of the subsamples. For CLL, both MDC and MCP‐1 were selected with proportions around 20%, and for DLBCL, sCD40L showed a selection proportion around 25%, while it was around 15% for MCP‐1.

## Discussion

In our study of plasma levels of circulating immune markers and subsequent risk of BCL and main histological subtypes, growth factors and in particular, FGF‐2, TGF‐α and VEGF were found consistently associated (inversely) with incidence of MM. These associations seemed to persist among cases sampled more than six years before diagnosis, and none of these disease‐associated inflammatory markers showed an association with time to diagnosis in cases only.

For CLL, DLBCL and FL, no significant association between circulating immune markers was observed in univariate analyses. However, adopting a group PLS approach, better accounting for the possible pleiotropic and complex effects of immune markers on BCL incidence, chemokines as a group were found to be related to DLBCL and cytokines to CLL. Additional variable selection within each selected groups identified individual markers driving the link between the group and the outcome: sCD40L and IL4 for CLL; and MDC, eotaxin, MCP‐1, and IP10 for DLBCL. These links were not detected using the univariate approach. These results indicate that the use of group and sparse‐group PLS may enhance the analyses of interrelated biological markers.

No direct evidence from our analyses provided support for the previously reported associations between TNF,^9^ IL13^8^ and IL10^9^, ^10^, ^14^ and BCL; TNF‐α,^11^ IL6^14^ and IL10^9^, ^14^ and FL; TNF‐α,^11^ IL5 and IL10^9^ and DLBCL and TGF‐α^11^ and TNF^9^ and CLL. However, the direction of most of the previously reported associations is consistent with our findings. In particular, as Gu *et al*.^8^ reported (among 92 B‐NHL cases and 184 matched controls), we find a decreased risk of B‐NHL with increasing levels of IL13 and IL5 and an increased risk of B‐NHL for TNF‐α. As previously reported in a study involving 491 B‐NHL cases and 491 controls,^9^ we also find a positive correlation between levels of TNF‐α, IL10 and the incidence of all BCL, FL and DLBCL. The associations reported by Purdue *et al*.^10^, ^11^ and Conroy *et al*.^14^ (272 NHL cases and 541 matched controls) linking TNF‐α and FL, TGF‐α and CLL and IL10 and BCL and FL were also in the same direction of ours. As such, our study does provide some potential meta‐analytical support for reported associations involving blood levels of immune markers and specific BCL‐subtypes.

Most previous prospective studies on immune markers and lymphoma did not include MM. In the pooled analyses of the MM Cohort Consortium included 493 MM cases and 978 controls from 8 cohorts, IGF‐1 was found associated with an increased MM risk within 3 years of blood collection while soluble IL‐6 receptor was associated with MM in the 6 first years after blood collection.^13^ Therefore, these markers are likely to reflect the tumor and/or its microenvironment. In contrast, our study revealed several markers, mostly growth factors, inversely related to long‐term risk of MM. This may be of importance as the average 5‐year survival rate for MM patients remains low (∼45%).^27^ If results of our study are replicated in other studies and extended to clinical studies in patients with monoclonal gammopathy of undetermined significance (MGUS), or smoldering MM (SMM), this could lead to the identification of patients at higher risk of progressing to MM, and, in the long‐term could improve individualized surveillance strategies.

We also performed sparse PLS analyses to identify (combination of) biomarkers that would be indicative of the time elapsed between protein measurement and clinical onset in a case‐only setting. These analyses revealed that GMCSF plasma levels were increasing closer to diagnosis for all BCL cases, and in particular MM cases. A clinically relevant aspect of the interactions of MM plasma cells in the bone marrow microenvironment is neovascularization, which is central in disease progression.^28^ Myeloma plasma cells induce angiogenesis *via* recruitment and activation of stromal inflammatory cells such as macrophages and mast cells. When these cells are activated, they secrete angiogenic factors including GM‐CSF, which contribute to enhance the tumor neovascularization.^29^ Recently GMCSF has also been found to be involved in homing circulating endothelial precursor cells, which contribute to the “angiogenic switch” and tumor progression.^29^

Our study has a number of strengths, including its prospective nature, which limits reverse causation bias that may occur when variation in blood level of cytokines is induced by the disease itself, cancer treatments or lifestyle changes after cancer diagnosis. Moreover, compared to most previous prospective studies, especially on MM, we measured a larger panel of immune markers. The availability of two cohorts allowed for independent confirmation of the observed signals. Conversely, different media for blood samples (citrate in EPIC‐Italy and EDTA in NSHDS) might have introduced differences in cytokine levels between the two cohorts which may cause bias in unconditional analyses by incomplete correction for cohort status in the model. Although the use of different anticoagulants results in absolute differences in levels of immune markers, correlations between measurements in split samples simultaneously treated with heparin, citrate and EDTA have shown to be highly correlated.^15^, ^30^ Similarly, bias may arise from cytokine measurements of study subjects in two phases despite adjustment in multivariate analyses. However, stratified analyses by cohort and phase showed overall similar trends for the identified markers despite reduced power in these analyses. Furthermore, FGF‐2, for which a significant association with future MM risk was found, presented a relatively low ICC (0.43). The consequence of a low ICC would be an underestimation of the effect and would not introduce a false‐positive association. As such, we may have underestimated the predictive power of FGF‐2 which warrants follow‐up in future studies.

We measured the immune markers at a single time point to determine future risk of BCL, which may not accurately reflect the long‐term immune status of an individual. However, several studies have provided evidence of a reasonable between‐to‐within person variability ratio (ICC) suggesting temporal stability for panels of cytokines.^30^, ^31^, ^32^, ^33^, ^34^, ^35^ Finally, blood cytokines are produced not only by those cell types considered to play pivotal roles in the immune system and in inflammatory responses, including lymphocytes, monocytes and mast cells but also by macrophages and, for some cytokines, also fibroblasts, neutrophils and endothelial cells. So, it should be noted that plasma level of cytokines may not necessarily reflect activity in the target tissue.

Our study provided evidence for a strong link between FGF‐2 and TGF‐α levels and incidence of MM. Several clinical studies have reported that the plasma concentrations of FGF‐2 were elevated in patients with active MM compared to patients with inactive disease, and this correlates with increased bone marrow angiogenesis and lymphangiogenesis.^36^, ^37^, ^38^, ^39^ MM patients who respond to chemotherapy (an immunosuppressed condition) show a significant decrease in serum FGF‐2 levels, whereas non‐responders do not.^39^ TGF‐α is an important mitogen that binds to the EGF receptor and has been studied in many other malignancies, but data on MM are limited and no prospective data are available.^40^, ^41^, ^42^ In the sgPLS analyses, we also observed some moderate support for an effect of VEGF on MM incidence. Similar to FGF‐2, clinical studies have shown that increased serum levels of VEGF are associated with more advanced disease stages and with poor prognosis in BCL and MM cases.^36^, ^37^, ^39^ VEGF and its ligands and receptors have a central role in physiological regulation of angiogenesis.^43^ Moreover, there is a growing list of nonvascular roles of VEGF including recruitment of inflammatory cells and autocrine and intracrine production of hematopoietic stem cells.^43^ A recent nested case–control study within the Prostate, Lung, Colorectal and Ovarian Cancer Screening Trial showed a significant association between elevated blood levels of soluble VEGFR‐2 (sVEGFR‐2) and risk of BCL.^11^ Although the biologic function of sVEGFR2 is unclear, it has been shown that sVEGFR‐2 binds the lymphangiogenic growth factor VEGF‐C and thus inhibits VEGF‐C‐induced activation of VEGFR‐3, consequently inhibiting lymphatic endothelial cell proliferation.^44^ On the other hand, sVEGFR‐2 can bind VEGF and may act as a VEGF inhibitor. These studies support a possible role of the growth factors (VEGF and FGF‐2) in the pathogenesis of MM. Given their interrelationship and cyclic response, more in‐depth monitoring of the VEGF, FGF‐2 growth factors and its soluble receptors is needed to clarify their possible pre‐diagnostic role in MM.

Although the markers identified in our study have been identified previously in clinical studies of MM or its precursor states (*i.e*., MGUS and SMM),^45^, ^46^, ^47^, ^48^, ^49^, ^50^ the direction of our findings is in general opposite to observations among subjects diagnosed with MM, where higher concentrations of these markers seems to be related to generally poorer disease outcome. The reason for this difference in direction of the effect is not known but may hint toward a preclinical deregulation of these important biological systems in subjects developing MM later in life which at the time of clinical manifestation reverse in overexpression. However, we cannot exclude the bias related to limited statistical power and design‐related sources of variability in our findings.

In conclusion, our study showed that several immune markers, in particular growth factors, are associated with MM incidence in preclinical blood samples taken many years before clinical diagnosis. In addition, we provide marginal support for some of the previous reported associations between several immune‐markers and subtypes of BCL, in particular chemokines being related to DLBCL and cytokines with CLL. In addition, we showed a consistent link between blood levels of GMCSF to time‐to‐diagnosis in all BCL and MM cases. These results need to be extended and replicated in independent prospective cohorts to clarify the relationship with BCL risk for these markers.

## Supporting information

Supplementary Tables
