# Supplementary material for: Pre‐diagnostic blood immune markers, incidence and progression of B‐cell lymphoma and multiple myeloma: Univariate and functionally informed multivariate analyses
Source: Int J Cancer. 2018 Apr 26;143(6):1335–47. doi: 10.1002/ijc.31536 (PMC6100111; doi:10.1002/ijc.31536)
Supplement: Supplementary file 1 — Supplementary Tables [file IJC-143-1335-s001.docx]

**Supplementary Table 1.** Limit of detections (LOD), percentage of detection, mean Coefficient of Variations (CV), and ICC for each protein assayed

| **Manufacturer's name** | **% Detected** | **LOD (pg/mL)** | **Intra-CV** | |  | **Inter-CV** | | **Intra-class Correlation Coefficient** |
| --- | --- | --- | --- | --- | --- | --- | --- | --- |
|  |  |  | Phase 1 | Phase 2 |  | Phase 1 | Phase 2 |  |
| **Eotaxin** | 99.3 | 1.2 | 4.14 | 5.66 |  | 10.08 | 15.44 | 0.87 |
| **MCP-1** | 99.7 | 0.73 | 3.97 | 5.75 |  | 1.99 | 11.39 | 0.64 |
| **MCP-3** | 62.9 | 1.79 | 29.87 | 4.59 |  | 3.05 | 9.13 | 0.73 |
| **MIP-1α** | 71.7 | 0.45 | 4.37 | 4.48 |  | 2.38 | 10.55 | 0.72 |
| **MIP-1β** | 88.2 | 1.75 | 12.02 | 5.33 |  | 2.35 | 13.48 | 0.63 |
| **MDC** | 99.6 | 2.25 | 7.43 | 5.60 |  | 5.40 | 12.11 | 0.16 |
| **Fractalkine** | 76.6 | 1.3 | 5.92 | 4.71 |  | 4.12 | 19.62 | 0.77 |
| **GRO** | 99.5 | 3.8 | 7.06 | 4.15 |  | 9.27 | 8.67 | 0.90 |
| **IP-10** | 99.1 | 2.81 | 2.94 | 4.95 |  | 21.75 | 14.39 | 0.92 |
| **IL-8** | 99.2 | 0.07 | 29.10 | 7.12 |  | 4.84 | 12.96 | 0.88 |
| **INF-α** | 45 | 1.54 | 42.02 | 4.79 |  | 16.10 | 10.75 | 0.94 |
| **IL-1β** | 62 | 0.08 | 25.27 | 5.23 |  | 19.87 | 12.67 | 0.89 |
| **IL-2** | 76.3 | 0.09 | 26.99 | 6.77 |  | 7.81 | 16.43 | 0.78 |
| **IL-4** | 77 | 0.11 | 14.00 | 7.11 |  | 12.86 | 12.99 | 0.94 |
| **IL-5** | 84.7 | 0.04 | 25.80 | 8.73 |  | 47.87 | 24.51 | 0.85 |
| **IL-6** | 86.1 | 0.02 | 14.44 | 9.24 |  | 50.07 | 18.85 | 0.89 |
| **IL-7** | 66.8 | 0.08 | 7.53 | 7.87 |  | 6.15 | 19.79 | 0.97 |
| **IL-10** | 84.1 | 0.1 | 28.41 | 6.35 |  | 23.79 | 13.12 | 0.98 |
| **IL-13** | 55.0 | 0.04 | 20.92 | 6.04 |  | 23.89 | 16.63 | 0.89 |
| **INF-γ** | 63.8 | 0.14 | 10.09 | 7.13 |  | 9.78 | 16.24 | 0.97 |
| **sCD40l** | 99.2 | 0.23 | 6.30 | 4.93 |  | 3.76 | 16.24 | 0.80 |
| **TNF-α** | 98.9 | 0.01 | 17.29 | 7.89 |  | 3.78 | 10.95 | 0.90 |
| **EGF** | 73.4 | 1.73 | 11.92 | 5.13 |  | 6.72 | 10.47 | 0.74 |
| **GCSF** | 86.1 | 0.71 | 18.07 | 6.36 |  | 28.68 | 18.18 | 0.94 |
| **GMCSF** | 73.8 | 0.08 | 17.25 | 6.85 |  | 55.03 | 10.19 | 0.86 |
| **TGF-α** | 61.6 | 0.28 | 10.81 | 7.14 |  | 5.21 | 16.40 | 0.75 |
| **VEGF** | 75.4 | 8.83 | 9.49 | 5.51 |  | 0.52 | 14.11 | 0.70 |
| **FGF2** | 68.6 | 2.21 | 18.36 | 6.76 |  | 6.20 | 20.45 | 0.43 |

**Supplementary Table 2**. Distribution of BCL subtypes and gender across phases and countries

|  | **Phase 1** | | **Phase 2** | |
| --- | --- | --- | --- | --- |
|  | **Italy** | **Sweden** | **Italy** | **Sweden** |
| **DLBCL** | 10 | 9 | 1 | 24 |
| **FL** | 14 | 5 | 6 | 14 |
| **CLL** | 5 | 7 | 6 | 24 |
| **MM** | 10 | 18 | 11 | 37 |
| **Others** | 11 | 7 | 10 | 39 |
|  | **50** | **46** | **34** | **138** |
| **Sex** |  |  |  |  |
| **Male** | 40 | 54 | 28 | 142 |
| **Female** | 60 | 38 | 40 | 134 |

Numbers indicate cases in the top panel (BCL subtypes) and cases plus controls in the sex panel.

**Supplementary Table 3.** Median (Minimum- Maximum) values of immune markers stratified by case-control status, country and phase

|  | **Case, n=268** | **Control, n=268** | **EPIC, n=168** | **NSHDS, n=368** | **Phase 1, n=192** | **Phase 2, n=344** |
| --- | --- | --- | --- | --- | --- | --- |
| **Eotaxin** | 242.21 (8.40-990.58) | 239.31 (11.51-1122.54) | 48.02 (8.40-319.46) | 333.12 (35.34-1122.54) | 156.82 (15.64-1122.54) | 255.09 (8.40-1093.35) |
| **MCP-1** | 279.41 (1.78-787.60) | 297.16 (6.20-1085.85) | 213.27 (55.08-634.85) | 323.49 (1.78-1085.85) | 304.35 (55.08-1085.85) | 278.89 (1.78-948.07) |
| **MCP-3** | 6.01 (0.02-1225.93) | 13.42 (0.08-1003.80) | 8.18 (0.02-1225.93) | 9.43 (0.02-1003.80) | 18.04 (0.14-1225.93) | 4.32 (0.02-914.30) |
| **MIP-1α** | 7.28 (0.01-398.74) | 12.17 (0.01-276.28) | 13.83 (0.09-398.74) | 8.33 (0.01-276.28) | 43.30 (0.69-398.74) | 4.49 (0.01-125.18) |
| **MIP-1β** | 30.84 (0.27-843.45) | 36.38 (0.28-1112.56) | 33.67 (1.99-572.67) | 33.12 (0.27-1112.56) | 48.03 (2.95-1112.56) | 25.16 (0.27-424.76) |
| **MDC** | 705.34 (42.59-8962.26) | 731.65 (50.85-7453.92) | 335.82 (50.85-3363.32) | 870.55 (42.59-8962.26) | 758.34 (91.40-8962.26) | 690.66 (42.59-4269.19) |
| **Fractalkine** | 36.62 (0.13-3229.59) | 56.81 (0.35-4599.65) | 82.54 (0.58-1923.42) | 30.46 (0.13-4599.65) | 118.39 (0.65-4599.65) | 23.14 (0.13-3229.59) |
| **GRO** | 391.31 (27.15-2188.12) | 369.11 (44.38-2914.05) | 216.50 (27.15-1860.16) | 426.08 (69.12-2914.05) | 553.45 (54.30-2914.05) | 338.73 (27.15-1319.35) |
| **IP-10** | 447.71 (15.86-3374.05) | 446.74 (8.88-3766.50) | 248.48 (59.19-1294.32) | 543.60 (8.88-3766.50) | 417.10 (81.68-3766.50) | 465.81 (8.88-2738.33) |
| **IL-8** | 4.03 (0.59-304.82) | 4.53 (0.67-190.86) | 9.75 (0.67-304.82) | 4.00 (0.59-167.32) | 9.75 (1.10-304.82) | 3.26 (0.59-59.12) |
| **INF-α** | 2.06 (0.0007-2569.21) | 4.69 (0.00003-1148.80) | 3.93 (0.002-1034.96) | 2.59 (0.00003-2569.21) | 1.37 (0.00003-2569.21) | 3.58 (0.002-885.96) |
| **IL-1β** | 0.44 (0.000001-435.52) | 0.96 (0.002-350.58) | 1.37 (0.000001-254.81) | 0.39 (0.00003-435.52) | 0.39 (0.000001-435.52) | 0.83 (0.01-350.58) |
| **IL-2** | 2.53 (0.03-627.98) | 3.04 (0.01-2224.85) | 11.55 (0.02-300.87) | 2.00 (0.01-2224.85) | 10.45 (0.01-300.87) | 1.97 (0.02-2224.85) |
| **IL-4** | 3.19 (0.02-564.88) | 6.54 (0.01-1627.27) | 0.73 (0.04-66.30) | 9.96 (0.01-1627.27) | 1.06 (0.02-1627.27) | 10.49 (0.01-776.61) |
| **IL-5** | 0.86 (0.00001-525.55) | 0.87 (0.02-332.06) | 2.31 (0.04-525.55) | 0.62 (0.00001-332.06) | 2.31 (0.009-525.55) | 0.63 (0.01-49.89) |
| **IL-6** | 3.77 (0.04-480.80) | 4.00 (0.04-1314.71) | 2.40 (0.12-45.99) | 5.09 (0.04-1314.71) | 4.77 (0.04-1314.71) | 3.53 (0.09-322.65) |
| **IL-7** | 0.65 (0.01-303.91) | 0.75 (0.01-417.49) | 1.73 (0.14-18.76) | 0.36 (0.01-417.49) | 1.60 (0.03-417.49) | 0.41 (0.01-140.64) |
| **IL-10** | 12.19 (0.10-1322.50) | 10.82 (0.07-2635.70) | 11.01 (0.07-297.55) | 11.66 (0.10-2635.70) | 15.50 (0.07-2635.70) | 9.21 (0.32-2333.34) |
| **IL-13** | 2.06 (0.01-929.40) | 3.98 (0.01-2474.46) | 2.72 (0.02-929.40) | 4.01 (0.01-2474.46) | 3.77 (0.01-2474.46) | 2.09 (0.02-562.68) |
| **INF-γ** | 1.36 (0.00-2069.35) | 2.20 (0.01-1591.65) | 1.43 (0.0003-2069.35) | 1.90 (0.0005-1591.65) | 1.70 (0.0003-2069.35) | 1.73 (0.02-1591.65) |
| **sCD40l** | 689.32 (12.33-7627.75) | 579.19 (4.39-5357.61) | 404.41 (4.39-2819.65) | 775.10 (12.33-7627.75) | 723.67 (4.39-7627.75) | 593.06 (12.33-3994.34) |
| **TNF-α** | 6.02 (0.30-111.78) | 5.11 (0.59-854.31) | 4.97 (0.81-38.00) | 5.85 (0.30-854.31) | 6.62 (0.81-38.00) | 4.96 (0.30-854.31) |
| **EGF** | 22.31 (0.02-1622.54) | 24.97 (0.14-1561.89) | 20.31 (0.15-1622.54) | 24.23 (0.02-842.01) | 40.28 (0.25-1622.54) | 19.81 (0.02-550.46) |
| **GCSF** | 24.47 (0.56-496.00) | 24.73 (0.77-2649.79) | 27.90 (0.56-304.12) | 23.04 (0.77-2649.79) | 28.23 (1.24-496.00) | 22.72 (0.56-2649.79) |
| **GMCSF** | 2.70 (0.02-285.46) | 2.80 (0.01-1566.28) | 4.91 (0.04-38.00) | 1.44 (0.01-1566.28) | 4.81 (0.03-281.84) | 1.41 (0.01-1566.28) |
| **TGF-α** | 1.10 (0.0007-1663.43) | 2.25 (0.01-842.46) | 1.46 (0.003-1663.43) | 1.69 (0.0007-842.46) | 9.08 (0.07-1663.43) | 0.83 (0.0007-399.24) |
| **VEGF** | 118.02 (0.46-6938.42) | 204.84 (0.47-5644.06) | 143.29 (0.81-4204.53) | 162.41 (0.46-6938.42) | 409.61 (2.98-6938.42) | 82.02 (0.46-2549.75) |
| **FGF2** | 15.44 (0.04-1969.88) | 27.12 (0.10-1005.98) | 25.71 (0.38-1969.88) | 18.84 (0.04-1005.98) | 42.09 (0.18-1969.88) | 13.79 (0.04-410.95) |

**Supplementary Table 4-A.** Median (Minimum- Maximum) values of immune markers stratified by BCL subtypes for EPIC –Italy cohort

|  | **DLBCL, n=11** | **FL, n=20** | **CLL, n=11** | **MM, n=21** |
| --- | --- | --- | --- | --- |
| **Eotaxin** | 53.16 (17.28-241.08) | 54.15 (8.40-118.72) | 42.22 (35.96-114.76) | 46.51 (22.17-111.48) |
| **MCP-1** | 184.53 (127.87-634.85) | 197.22 (98.66-351.39) | 216.80 (154.03-454.23) | 223.55 (140.45-549.76) |
| **MCP-3** | 39.28 (1.79-192.25) | 10.48 (0.14-165.72) | 5.27 (0.60-734.54) | 5.86 (0.09-83.15) |
| **MIP-1α** | 20.31 (1.25-398.74) | 26.56 (0.27-140.62) | 8.72 (0.27-82.86) | 2.64 (0.14-210.96) |
| **MIP-1β** | 31.50 (20.67-58.48) | 37.45 (10.57-174.02) | 26.73 (2.61-336.15) | 23.18 (1.99-119.93) |
| **MDC** | 355.73 (91.40-2142.34) | 328.66 (80.47-1065.02) | 232.30 (94.77-1252.65) | 245.16 (103.41-2570.29) |
| **Fractalkine** | 117.74 (39.75-870.06) | 100.42 (6.69-525.23) | 71.64 (12.16-373.32) | 71.38 (0.98-1564.23) |
| **GRO** | 313.71 (115.07-524.73) | 269.72 (81.05-1343.18) | 145.04 (27.15-1789.58) | 199.54 (61.54-569.37) |
| **IP-10** | 395.74(111.74-1294.32) | 237.00 (74.09-449.82) | 231.11 (195.79-818.51) | 231.22 (86.73-459.02) |
| **IL-8** | 16.34 (1.50-304.82) | 11.18 (1.12-162.49) | 2.93 (0.72-123.57) | 4.01 (1.51-120.25) |
| **INF-α** | 0.28 (0.003-394.49) | 3.95 (0.002-673.94) | 13.90 (0.25-362.53) | 3.79 (0.01-81.92) |
| **IL-1β** | 4.34 (0.03-163.78) | 1.33 (0.000001-17.46) | 0.37 (0.02-10.24) | 1.35 (0.05-7.73) |
| **IL-2** | 16.67 (1.20-69.11) | 19.63 (0.13-224.77) | 1.07 (0.09-166.80) | 3.44 (0.09-83.17) |
| **IL-4** | 0.38 (0.08-3.16) | 0.75 (0.07-22.22) | 0.47 (0.23-4.23) | 1.07 (0.17-59.30) |
| **IL-5** | 3.19 (0.17-75.84) | 4.91 (0.10-39.69) | 0.33 (0.08-525.55) | 1.34 (0.11-49.96) |
| **IL-6** | 2.26 (0.19-45.99) | 3.32 (0.23-29.17) | 1.22 (0.28-27.67) | 2.10 (0.13-10.44) |
| **IL-7** | 1.94 (0.74-4.86) | 2.22 (0.14-6.46) | 1.21 (0.22-18.76) | 1.40 (0.34-5.50) |
| **IL-10** | 4.70 (0.18-266.23) | 13.40 (0.18-161.99) | 9.24 (0.86-297.55) | 11.39 (0.26-88.63) |
| **IL-13** | 10.61.(0.53-33.85) | 3.48 (0.02-140.31) | 1.57 (0.23-929.40) | 1.52 (0.07-44.11) |
| **INF-γ** | 1.99 (0.04-57.33) | 1.48 (0.0003-34.10) | 0.54 (0.11-2069.35) | 0.86 (0.01-25.99) |
| **sCD40l** | 398.44 (143.41-2819.65) | 390.52 (282.82-2339.77) | 383.16 (110.35-1429.72) | 431.83 (155.27-2027.04) |
| **TNF-α** | 4.71 (0.81-12.18) | 6.02 (1.64-20.45) | 6.20 (2.44-12.44) | 5.56 (1.33-10.18) |
| **EGF** | 27.58 (0.35-305.56) | 28.25 (0.25-211.41) | 25.95 (1.47-265.24) | 11.57 (0.15-338.79) |
| **GCSF** | 31.10 (18.17-54.55) | 30.10 (4.20-304.12) | 31.99 (6.61-101.51) | 28.29 (0.56-104.80) |
| **GMCSF** | 4.71 (0.81-12.18) | 5.32 (0.15-20.45) | 1.91 (0.11-10.47) | 5.96 (0.19-24.01) |
| **TGF-α** | 2.55 (0.60-81.12) | 2.47 (0.003-1663.43) | 0.39 (0.06-17.06) | 0.78 (0.04-185.92) |
| **VEGF** | 203.09 (8.33-2875.48) | 231.39 (1.76-2346.19) | 240.55 (3.27-1245.17) | 23.37 (0.81-2578.85) |
| **FGF2** | 73.94 (4.27-222.42) | 19.95 (0.91-359.63) | 23.76 (1.00-836.47) | 8.83 (0.99-390.82) |

**Supplementary Table 4-B.** Median (Minimum- Maximum) values of immune markers stratified by BCL subtypes for NSHDS cohort

|  | **DLBCL, n=33** | **FL, n=19** | **CLL, n=31** | **MM, n=55** |
| --- | --- | --- | --- | --- |
| **Eotaxin** | 351.77 (142.96-985.52) | 329.76 (152.89-990.58) | 373.04 (192.63-813.23) | 341.53 (95.61-944.76) |
| **MCP-1** | 325.70 (17.17-689.56) | 306.84 (2.20-747.54) | 355.69 (70.58-787.60) | 346.09 (1.78-786.86) |
| **MCP-3** | 9.43 (0.08-914.30) | 10.69 (0.36-70.88) | 7.92 (0.37-121.48) | 2.30 (0.02-312.82) |
| **MIP-1α** | 8.87 (0.05-124.70) | 7.66 (0.01-230.61) | 10.83 (0.20-100.73) | 5.88 (0.01-207.93) |
| **MIP-1β** | 37.00 (2.52-273.28) | 28.61 (1.07-702.97) | 36.69 (2.53-285.75) | 30.85 (0.27-843.45) |
| **MDC** | 921.91 (42.59-2493.68) | 873.91 (214.98-5878.03) | 904.71 (160.02-8962.26) | 889.71 (170.88-6259.24) |
| **Fractalkine** | 35.54 (0.58-1260.43) | 42.90 (0.66-1347.33) | 22.85 (2.15-2927.00) | 19.22 (0.13-3229.59) |
| **GRO** | 435.02 (134.68-2188.12) | 358.68 (102.64-1782.63) | 442.67 (138.7-1534.08) | 475.63 (78.81-1748.74) |
| **IP-10** | 532.43 (151.00-2021.13) | 534.50 (38.07-1959.53) | 535.40 (192.1-1648.62) | 588.06 (15.86-3374.05) |
| **IL-8** | 4.22 (0.89-59.12) | 2.81 (0.69-9.80) | 3.10 (0.59-29.57) | 3.83 (0.64-41.85) |
| **INF-α** | 2.67 (0.002-778.15) | 2.97 (0.01-2569.21) | 2.39 (0.003-670.65) | 1.54 (0.001-645.33) |
| **IL-1β** | 0.41 (0.01-199.02) | 0.24 (0.01-14.17) | 0.18 (0.01-435.52) | 0.28 (0.00003-21.24) |
| **IL-2** | 1.96 (0.24-282.42) | 2.33 (0.20-47.92) | 0.85 (0.04-201.86) | 1.04 (0.07-123.28) |
| **IL-4** | 9.14 (0.02-274.25) | 6.70 (0.53-184.39) | 1.19 (0.12-435.84) | 8.67 (0.02-372.43) |
| **IL-5** | 0.72 (0.03-40.95) | 0.27 (0.06-35.55) | 0.25 (0.03-49.89) | 0.63 (0.01-29.25) |
| **IL-6** | 4.93 (0.04-133.18) | 2.63 (0.38-480.80) | 2.33 (0.09-350.99) | 4.64 (0.04-46.45) |
| **IL-7** | 0.36 (0.01-64.42) | 0.51 (0.02-303.91) | 0.29 (0.03-81.26) | 0.35 (0.05-39.07) |
| **IL-10** | 13.59 (1.50-1322.50) | 11.57 (2.15-688.93) | 7.44 (0.10-479.88) | 10.61 (0.26-130.10) |
| **IL-13** | 4.05 (0.12-204.70) | 0.88 (0.02-178.38) | 1.19 (0.09-669.04) | 1.09 (0.01-173.84) |
| **INF-γ** | 2.37 (0.02-619.75) | 1.74 (0.28-106.09) | 1.87 (0.02-281.84) | 1.26 (0.001-168.82) |
| **sCD40l** | 709.77 (22.96-6393.33) | 859.35 (92.92-1857.87) | 937.92 (189.1-3994.34) | 858.07 (32.53-3807.63) |
| **TNF-α** | 6.56 (0.85-81.53) | 4.98 (0.96-34.57) | 7.44 (1.00-35.58) | 6.90 (1.22-24.74) |
| **EGF** | 18.42 (0.02-129.72) | 23.32 (0.67-241.93) | 23.16 (1.24-275.71) | 19.24 (0.25-842.01) |
| **GCSF** | 17.79 (1.08-496.00) | 23.23 (2.96-57.51) | 23.77 (1.45-363.57) | 24.40 (1.16-303.00) |
| **GMCSF** | 3.13 (0.11-265.24) | 1.12 (0.07-44.77) | 0.64 (0.05-281.84) | 1.48 (0.02-33.61) |
| **TGF-α** | 0.85 (0.001-290.88) | 2.23 (0.01-267.82) | 1.16 (0.06-100.19) | 0.98 (0.002-254.11) |
| **VEGF** | 118.53 (2.99-2218.52) | 234.04 (5.37-5962.78) | 261.49 (4.98-2520.86) | 84.10 (0.46-6938.42) |
| **FGF2** | 20.08 (0.39-236.19) | 23.59 (0.30-309.99) | 21.90 (0.58-195.91) | 7.60 (0.04-483.71) |

**Supplementary Table 5.** **Results of the mixed model relating log-transformed concentrations of immune markers and all B-cell lymphoma cases.** Results are presented for all BCL (N=268 case control pairs) and for each histological subtype separately.

|  | **All BCL** | | |  | | **CLL** | | |  | | **DLBCL** | | |  | | **FL** | | |  | | **MM** | |
| --- | --- | --- | --- | --- | --- | --- | --- | --- | --- | --- | --- | --- | --- | --- | --- | --- | --- | --- | --- | --- | --- | --- |
|  | *β* | *p-value* |  | | *β* | | *p-value* |  | | *β* | | *p-value* |  | | *β* | | *p-value* |  | | *β* | | *p-value* |
| EGF | -0.28 | 5.25E-02 |  | | -0.05 | | 8.30E-01 |  | | -0.53 | | 6.18E-02 |  | | -0.19 | | 5.24E-01 |  | | -0.53 | | 1.73E-02 |
| Eotaxin | 0.05 | 2.64E-01 |  | | 0.12 | | 1.45E-01 |  | | 0.13 | | 1.31E-01 |  | | 0.02 | | 8.03E-01 |  | | 0.03 | | 6.59E-01 |
| FGF2 | **-0.50** | **7.22E-04** |  | | -0.17 | | 5.39E-01 |  | | -0.10 | | 7.22E-01 |  | | -0.32 | | 2.61E-01 |  | | **-1.10** | | **7.80E-07** |
| Fractalkine | -0.31 | 2.80E-02 |  | | -0.17 | | 5.36E-01 |  | | 0.01 | | 9.57E-01 |  | | -0.01 | | 9.61E-01 |  | | **-0.72** | | **1.12E-03** |
| GCSF | -0.10 | 2.58E-01 |  | | -0.14 | | 4.37E-01 |  | | -0.06 | | 7.24E-01 |  | | 0.01 | | 9.55E-01 |  | | -0.13 | | 3.74E-01 |
| GRO | 0.03 | 5.11E-01 |  | | 0.02 | | 8.46E-01 |  | | 0.07 | | 4.11E-01 |  | | -0.04 | | 6.51E-01 |  | | 0.02 | | 7.29E-01 |
| INFa | -0.42 | 8.42E-02 |  | | -0.02 | | 9.62E-01 |  | | -0.82 | | 8.83E-02 |  | | 0.09 | | 8.55E-01 |  | | -0.68 | | 6.71E-02 |
| IP10 | 0.03 | 6.44E-01 |  | | 0.12 | | 2.34E-01 |  | | 0.04 | | 6.78E-01 |  | | -0.03 | | 7.51E-01 |  | | 0.04 | | 5.91E-01 |
| MCP1 | -0.01 | 8.81E-01 |  | | 0.15 | | 1.46E-01 |  | | 0.03 | | 7.67E-01 |  | | -0.11 | | 3.30E-01 |  | | 0.01 | | 8.91E-01 |
| MCP3 | -0.40 | 1.11E-02 |  | | -0.31 | | 2.97E-01 |  | | -0.01 | | 9.66E-01 |  | | -0.28 | | 3.26E-01 |  | | **-0.91** | | **1.36E-04** |
| MDC | -0.16 | 3.04E-03 |  | | -0.08 | | 4.42E-01 |  | | -0.26 | | 1.81E-02 |  | | -0.09 | | 3.79E-01 |  | | -0.19 | | 1.61E-02 |
| MIP1a | -0.35 | 8.33E-03 |  | | 0.10 | | 7.19E-01 |  | | -0.15 | | 5.63E-01 |  | | -0.31 | | 2.34E-01 |  | | **-0.72** | | **4.60E-04** |
| MIP1b | -0.16 | 1.18E-01 |  | | 0.04 | | 8.46E-01 |  | | -0.12 | | 5.58E-01 |  | | -0.03 | | 8.93E-01 |  | | -0.44 | | 5.91E-03 |
| sCD40L | 0.19 | 8.88E-03 |  | | 0.35 | | 1.42E-02 |  | | 0.19 | | 1.86E-01 |  | | 0.26 | | 7.27E-02 |  | | 0.10 | | 3.40E-01 |
| TGFa | **-0.68** | **6.55E-05** |  | | -0.66 | | 3.71E-02 |  | | -0.49 | | 1.44E-01 |  | | -0.46 | | 1.80E-01 |  | | **-1.08** | | **4.08E-05** |
| VEGF | -0.42 | 8.60E-03 |  | | 0.12 | | 6.86E-01 |  | | -0.33 | | 2.72E-01 |  | | -0.09 | | 7.81E-01 |  | | **-1.00** | | **4.23E-05** |
| IL1b | -0.27 | 7.31E-02 |  | | -0.49 | | 7.25E-02 |  | | 0.42 | | 1.18E-01 |  | | -0.29 | | 3.12E-01 |  | | -0.59 | | 5.48E-03 |
| IL2 | -0.05 | 7.21E-01 |  | | -0.07 | | 7.64E-01 |  | | 0.13 | | 6.12E-01 |  | | 0.21 | | 3.99E-01 |  | | -0.23 | | 2.34E-01 |
| IL4 | -0.15 | 2.76E-01 |  | | -0.68 | | 1.28E-02 |  | | -0.32 | | 2.61E-01 |  | | -0.07 | | 7.88E-01 |  | | -0.02 | | 9.36E-01 |
| IL5 | -0.10 | 3.95E-01 |  | | -0.22 | | 2.81E-01 |  | | 0.20 | | 3.54E-01 |  | | -0.02 | | 9.29E-01 |  | | -0.08 | | 6.36E-01 |
| IL6 | -0.03 | 7.89E-01 |  | | -0.15 | | 4.87E-01 |  | | -0.14 | | 5.53E-01 |  | | -0.03 | | 8.89E-01 |  | | -0.22 | | 1.92E-01 |
| IL7 | 0.07 | 5.36E-01 |  | | -0.22 | | 3.61E-01 |  | | 0.11 | | 6.56E-01 |  | | 0.28 | | 2.58E-01 |  | | -0.08 | | 6.69E-01 |
| IL8 | -0.08 | 2.84E-01 |  | | -0.15 | | 2.58E-01 |  | | 0.04 | | 7.63E-01 |  | | -0.07 | | 5.99E-01 |  | | -0.12 | | 2.33E-01 |
| IL10 | 0.12 | 3.04E-01 |  | | -0.09 | | 7.08E-01 |  | | 0.27 | | 2.69E-01 |  | | 0.30 | | 2.33E-01 |  | | -0.07 | | 7.12E-01 |
| IL13 | -0.03 | 8.51E-01 |  | | -0.13 | | 6.75E-01 |  | | 0.30 | | 3.10E-01 |  | | 0.14 | | 6.66E-01 |  | | -0.52 | | 3.85E-02 |
| INFg | -0.31 | 8.04E-02 |  | | -0.16 | | 6.25E-01 |  | | 0.05 | | 8.75E-01 |  | | -0.04 | | 9.11E-01 |  | | -0.75 | | 6.19E-03 |
| GMCSF | -0.07 | 5.84E-01 |  | | -0.30 | | 2.21E-01 |  | | 0.30 | | 2.32E-01 |  | | 0.05 | | 8.37E-01 |  | | -0.21 | | 2.86E-01 |
| TNFa | 0.12 | 6.60E-02 |  | | 0.13 | | 3.07E-01 |  | | 0.13 | | 2.83E-01 |  | | 0.08 | | 5.13E-01 |  | | 0.12 | | 1.88E-01 |

IL indicates Interleukin; INF-α, interferon alpha; INF-γ, interferon gamma; GMCSF, Granulocyte-macrophage colony stimulating factor; TNF-α, Tumor necrosis factor alpha; EGF, Epidermal growth factor; FGF-2, Fibroblast growth factor 2; GCSF, Granulocyte colony-stimulating factor; GRO, melanoma growth stimulatory activity/growth-related oncogene; IP10, Interferon gamma-induced protein 10; MCP-1, Monocyte chemotactic protein-1; MCP-3, Monocyte chemotactic protein-3; MDC, Macrophage Derived Chemokine; MIP-1α, Macrophage Inflammatory Protein 1 alpha; MIP-1ß, Macrophage Inflammatory Protein 1 beta; sCD40L, Soluble CD40 ligand; VEGF, vascular endothelial growth factor; and TGF-α, Transforming growth factor alpha; CLL, chronic lymphocytic leukemia; DLBCL, diffuse large B-cell lymphoma; FL, follicular lymphoma; MM, multiple myeloma; Models were adjusted for age, gender, country, experimental phase, body mass index, education, physical activity, smoking status at enrolment, and alcohol intake at enrolment .

**Supplementary Table 6.** **Results of the mixed model relating log-transformed concentrations of immune markers and all B-cell lymphoma cases and main histological subtypes adjusted for the estimated white blood cell compositions (N=224 case control pairs).** Results are presented for all BCL and for each histological subtype separately and models are adjusted for estimated blood proportions of CD8, CD4, Natural Killer T cells, B cells, and monocytes.

|  | **All BCL** | | |  | | **CLL** | | |  | | **DLBCL** | | |  | | **FL** | | |  | | **MM** | |
| --- | --- | --- | --- | --- | --- | --- | --- | --- | --- | --- | --- | --- | --- | --- | --- | --- | --- | --- | --- | --- | --- | --- |
|  | *β* | *p-value* |  | | *β* | | *p-value* |  | | *β* | | *p-value* |  | | *β* | | *p-value* |  | | *β* | | *p-value* |
| EGF | -0.36 | 2.57E-02 |  | | -0.01 | | 9.60E-01 |  | | -0.48 | | 1.08E-01 |  | | -0.12 | | 7.20E-01 |  | | -0.58 | | 1.71E-02 |
| Eotaxin | 0.03 | 4.59E-01 |  | | 0.05 | | 5.93E-01 |  | | 0.19 | | 3.42E-02 |  | | 0.01 | | 9.27E-01 |  | | 0.03 | | 6.17E-01 |
| FGF2 | **-0.52** | **1.23E-03** |  | | -0.08 | | 7.84E-01 |  | | 0.06 | | 8.42E-01 |  | | -0.25 | | 4.30E-01 |  | | **-1.20** | | **4.25E-07** |
| Fractalkine | -0.47 | 1.84E-03 |  | | -0.19 | | 5.56E-01 |  | | 0.16 | | 5.61E-01 |  | | 0.02 | | 9.69E-01 |  | | **-1.03** | | **1.08E-05** |
| GCSF | -0.13 | 1.76E-01 |  | | -0.27 | | 2.02E-01 |  | | -0.06 | | 7.54E-01 |  | | 0.07 | | 7.12E-01 |  | | -0.14 | | 3.44E-01 |
| GRO | 0.01 | 8.50E-01 |  | | -0.02 | | 8.23E-01 |  | | 0.11 | | 2.29E-01 |  | | -0.06 | | 5.55E-01 |  | | 0.05 | | 5.41E-01 |
| INFa | -0.47 | 8.05E-02 |  | | 0.32 | | 5.83E-01 |  | | -0.71 | | 1.73E-01 |  | | 0.12 | | 8.30E-01 |  | | -0.95 | | 1.75E-02 |
| IP10 | 0.02 | 7.77E-01 |  | | 0.05 | | 7.07E-01 |  | | 0.07 | | 4.96E-01 |  | | 0.03 | | 7.92E-01 |  | | 0.08 | | 3.97E-01 |
| MCP1 | 0.02 | 7.13E-01 |  | | 0.17 | | 1.89E-01 |  | | 0.05 | | 6.40E-01 |  | | -0.04 | | 7.22E-01 |  | | 0.11 | | 2.30E-01 |
| MCP3 | -0.42 | 1.29E-02 |  | | -0.19 | | 5.87E-01 |  | | 0.27 | | 3.76E-01 |  | | -0.18 | | 5.52E-01 |  | | **-1.15** | | **6.20E-06** |
| MDC | -0.15 | 1.15E-02 |  | | -0.06 | | 6.34E-01 |  | | -0.13 | | 2.62E-01 |  | | -0.04 | | 7.22E-01 |  | | -0.19 | | 2.41E-02 |
| MIP1a | -0.36 | 1.27E-02 |  | | 0.01 | | 9.87E-01 |  | | 0.02 | | 9.71E-01 |  | | -0.12 | | 6.51E-01 |  | | **-0.74** | | **8.91E-04** |
| MIP1b | -0.17 | 1.37E-01 |  | | 0.05 | | 8.55E-01 |  | | 0.01 | | 9.39E-01 |  | | -0.01 | | 9.57E-01 |  | | -0.48 | | 5.77E-03 |
| sCD40L | 0.19 | 1.92E-02 |  | | 0.38 | | 2.93E-02 |  | | 0.21 | | 1.81E-01 |  | | 0.25 | | 1.21E-01 |  | | 0.09 | | 4.54E-01 |
| TGFa | **-0.76** | **4.99E-05** |  | | -0.89 | | 2.04E-02 |  | | -0.39 | | 2.77E-01 |  | | -0.41 | | 2.66E-01 |  | | **-1.15** | | **3.21E-05** |
| VEGF | -0.44 | 9.39E-03 |  | | 0.27 | | 4.53E-01 |  | | -0.20 | | 5.36E-01 |  | | 0.01 | | 9.68E-01 |  | | **-1.15** | | **8.23E-06** |
| IL1b | -0.32 | 5.92E-02 |  | | -0.49 | | 1.44E-01 |  | | 0.52 | | 7.44E-02 |  | | -0.42 | | 1.75E-01 |  | | **-0.71** | | **1.71E-03** |
| IL2 | -0.11 | 4.53E-01 |  | | 0.08 | | 8.06E-01 |  | | 0.18 | | 4.98E-01 |  | | 0.19 | | 4.94E-01 |  | | -0.40 | | 5.81E-02 |
| IL4 | -0.19 | 2.00E-01 |  | | -0.68 | | 4.18E-02 |  | | -0.24 | | 4.23E-01 |  | | -0.18 | | 5.55E-01 |  | | -0.18 | | 4.55E-01 |
| IL5 | -0.13 | 3.16E-01 |  | | 0.00 | | 9.89E-01 |  | | 0.33 | | 1.63E-01 |  | | 0.01 | | 9.59E-01 |  | | -0.31 | | 9.02E-02 |
| IL6 | -0.03 | 7.95E-01 |  | | -0.21 | | 4.27E-01 |  | | 0.01 | | 9.73E-01 |  | | 0.04 | | 8.89E-01 |  | | -0.37 | | 4.79E-02 |
| IL7 | 0.06 | 6.40E-01 |  | | -0.12 | | 6.81E-01 |  | | 0.24 | | 3.51E-01 |  | | 0.38 | | 1.60E-01 |  | | -0.26 | | 1.70E-01 |
| IL8 | -0.09 | 2.90E-01 |  | | -0.12 | | 4.67E-01 |  | | 0.14 | | 3.38E-01 |  | | -0.01 | | 9.28E-01 |  | | -0.18 | | 1.21E-01 |
| IL10 | 0.14 | 2.93E-01 |  | | -0.11 | | 7.00E-01 |  | | 0.32 | | 2.13E-01 |  | | 0.35 | | 1.99E-01 |  | | -0.16 | | 4.24E-01 |
| IL13 | -0.09 | 6.16E-01 |  | | 0.16 | | 6.72E-01 |  | | 0.40 | | 2.20E-01 |  | | 0.00 | | 9.91E-01 |  | | -0.82 | | 2.73E-03 |
| INFg | -0.40 | 4.26E-02 |  | | 0.02 | | 9.47E-01 |  | | 0.16 | | 6.32E-01 |  | | -0.02 | | 9.56E-01 |  | | **-0.96** | | **1.03E-03** |
| GMCSF | -0.08 | 5.39E-01 |  | | -0.05 | | 8.71E-01 |  | | 0.29 | | 2.77E-01 |  | | 0.16 | | 5.51E-01 |  | | -0.31 | | 1.30E-01 |
| TNFa | 0.12 | 8.34E-02 |  | | 0.10 | | 5.73E-01 |  | | 0.19 | | 1.68E-01 |  | | 0.13 | | 3.72E-01 |  | | 0.08 | | 4.23E-01 |

IL indicates Interleukin; INF-α, interferon alpha; INF-γ, interferon gamma; GMCSF, Granulocyte-macrophage colony stimulating factor; TNF-α, Tumor necrosis factor alpha; EGF, Epidermal growth factor; FGF-2, Fibroblast growth factor 2; GCSF, Granulocyte colony-stimulating factor; GRO, melanoma growth stimulatory activity/growth-related oncogene; IP10, Interferon gamma-induced protein 10; MCP-1, Monocyte chemotactic protein-1; MCP-3, Monocyte chemotactic protein-3; MDC, Macrophage Derived Chemokine; MIP-1α, Macrophage Inflammatory Protein 1 alpha; MIP-1ß, Macrophage Inflammatory Protein 1 beta; sCD40L, Soluble CD40 ligand; VEGF, vascular endothelial growth factor; and TGF-α, Transforming growth factor alpha CLL, chronic lymphocytic leukemia; DLBCL, diffuse large B-cell lymphoma; FL, follicular lymphoma; MM, multiple myeloma; Models were adjusted for age, gender, country, experimental phase, body mass index, education, physical activity, smoking status at enrolment, alcohol intake at enrolment , and WBC.

**Supplementary Table 7-A.** **Results of the mixed model relating log-transformed concentrations of immune markers and all B-cell lymphoma cases and main histological subtypes for samples from Phase 1 (96 pairs).**

|  | **All BCL** | |  | **CLL** | |  | **DLBCL** | |  | **FL** | |  | **MM** | |
| --- | --- | --- | --- | --- | --- | --- | --- | --- | --- | --- | --- | --- | --- | --- |
|  | *β* | *p-value* |  | *β* | *p-value* |  | *β* | *p-value* |  | *β* | *p-value* |  | *β* | *p-value* |
| EGF | -0.63 | 1.25e-02 |  | -0.1 | 8.27e-01 |  | -0.8 | 6.44e-02 |  | -0.55 | 2.12e-01 |  | -0.86 | 2.92e-02 |
| Eotaxin | 0.01 | 8.67e-01 |  | -0.17 | 3.11e-01 |  | 0.1 | 5.77e-01 |  | -0.03 | 8.48e-01 |  | -0.04 | 7.26e-01 |
| FGF2 | -0.69 | 2.52e-03 |  | -0.41 | 3.88e-01 |  | -0.29 | 4.86e-01 |  | -0.72 | 5.64e-02 |  | -1.23 | 7.16e-04 |
| Fractalkine | -0.25 | 2.09e-01 |  | -0.11 | 7.87e-01 |  | -0.42 | 2.80e-01 |  | 0.06 | 8.33e-01 |  | -0.49 | 1.20e-01 |
| GCSF | 0.07 | 5.12e-01 |  | -0.14 | 5.35e-01 |  | 0.02 | 8.93e-01 |  | 0.06 | 7.24e-01 |  | 0.12 | 5.06e-01 |
| GRO | 0.01 | 9.91e-01 |  | 0.04 | 9.31e-01 |  | 0.11 | 4.92e-01 |  | -0.02 | 9.02e-01 |  | -0.15 | 2.33e-01 |
| INFa | -0.66 | 1.72e-01 |  | 0.5 | 6.49e-01 |  | -2.03 | 2.60e-02 |  | 0.22 | 7.79e-01 |  | -1.71 | 2.65e-02 |
| IP10 | 0.1 | 2.01e-01 |  | 0.05 | 7.21e-01 |  | 0.16 | 2.26e-01 |  | 0 | 9.86e-01 |  | 0.26 | 2.96e-02 |
| MCP1 | 0.05 | 3.68e-01 |  | 0.01 | 9.53e-01 |  | 0.07 | 4.59e-01 |  | 0.04 | 6.87e-01 |  | 0.16 | 5.78e-02 |
| MCP3 | -0.61 | 8.13e-03 |  | -0.3 | 5.31e-01 |  | -0.27 | 5.24e-01 |  | -0.57 | 1.16e-01 |  | -1 | 5.70e-03 |
| MDC | -0.23 | 1.93e-02 |  | -0.34 | 1.07e-01 |  | -0.38 | 4.23e-02 |  | -0.14 | 4.00e-01 |  | -0.28 | 6.22e-02 |
| MIP1a | -0.64 | 4.24e-04 |  | -0.83 | 1.96e-02 |  | -0.49 | 1.34e-01 |  | -0.31 | 2.85e-01 |  | -0.9 | 1.26e-03 |
| MIP1b | -0.17 | 2.45e-01 |  | -0.21 | 5.23e-01 |  | -0.19 | 4.91e-01 |  | 0.02 | 9.19e-01 |  | -0.5 | 4.52e-02 |
| sCD40L | 0.27 | 3.19e-02 |  | 0.62 | 2.71e-02 |  | 0.18 | 4.48e-01 |  | 0.31 | 1.46e-01 |  | -0.06 | 8.02e-01 |
| TGFa | -1.17 | 4.62e-05 |  | -1.23 | 3.56e-02 |  | -0.63 | 2.20e-01 |  | -0.59 | 2.45e-01 |  | -1.73 | 1.41e-04 |
| VEGF | -0.57 | 2.95e-02 |  | -0.44 | 3.78e-01 |  | -0.37 | 4.36e-01 |  | -0.07 | 9.05e-01 |  | -1.13 | 7.68e-03 |
| IL1b | -0.49 | 1.17e-01 |  | -0.74 | 2.45e-01 |  | 0.53 | 2.67e-01 |  | -0.61 | 2.12e-01 |  | -1.53 | 4.05e-04 |
| IL2 | -0.01 | 9.59e-01 |  | 0.36 | 4.32e-01 |  | -0.54 | 1.44e-01 |  | 0.43 | 2.20e-01 |  | -0.71 | 3.34e-02 |
| IL4 | -0.36 | 1.24e-01 |  | -0.99 | 5.12e-02 |  | -0.67 | 1.26e-01 |  | -0.02 | 9.56e-01 |  | -0.53 | 1.49e-01 |
| IL5 | -0.13 | 5.24e-01 |  | -0.16 | 7.21e-01 |  | 0.09 | 8.12e-01 |  | 0.14 | 6.78e-01 |  | -0.67 | 2.78e-02 |
| IL6 | -0.09 | 7.01e-01 |  | -0.01 | 9.78e-01 |  | -0.54 | 2.15e-01 |  | 0.43 | 2.70e-01 |  | -0.82 | 2.19e-02 |
| IL7 | 0.1 | 6.36e-01 |  | -0.23 | 6.22e-01 |  | -0.18 | 6.03e-01 |  | 0.59 | 8.75e-02 |  | -0.52 | 8.14e-02 |
| IL8 | 0.02 | 8.96e-01 |  | 0.17 | 5.52e-01 |  | -0.02 | 9.55e-01 |  | 0.01 | 9.61e-01 |  | -0.28 | 1.53e-01 |
| IL10 | 0.22 | 4.05e-01 |  | -0.04 | 9.13e-01 |  | 0.19 | 6.95e-01 |  | 0.55 | 2.14e-01 |  | -0.55 | 1.46e-01 |
| IL13 | -0.01 | 9.79e-01 |  | -0.24 | 7.46e-01 |  | 0.4 | 4.85e-01 |  | 0.53 | 3.53e-01 |  | -1.62 | 1.85e-03 |
| INFg | -0.54 | 1.51e-01 |  | 0.68 | 3.73e-01 |  | -0.02 | 9.98e-01 |  | 0.02 | 9.71e-01 |  | -1.81 | 2.89e-03 |
| GMCSF | 0.04 | 8.24e-01 |  | 0.05 | 9.05e-01 |  | 0.11 | 7.39e-01 |  | 0.21 | 5.11e-01 |  | -0.56 | 7.36e-02 |
| TNFa | 0.13 | 1.12e-01 |  | 0.24 | 1.31e-01 |  | 0 | 9.93e-01 |  | 0.15 | 2.52e-01 |  | -0.07 | 5.28e-01 |

IL indicates Interleukin; INF-α, interferon alpha; INF-γ, interferon gamma; GMCSF, Granulocyte-macrophage colony stimulating factor; TNF-α, Tumor necrosis factor alpha; EGF, Epidermal growth factor; FGF-2, Fibroblast growth factor 2; GCSF, Granulocyte colony-stimulating factor; GRO, melanoma growth stimulatory activity/growth-related oncogene; IP10, Interferon gamma-induced protein 10; MCP-1, Monocyte chemotactic protein-1; MCP-3, Monocyte chemotactic protein-3; MDC, Macrophage Derived Chemokine; MIP-1α, Macrophage Inflammatory Protein 1 alpha; MIP-1ß, Macrophage Inflammatory Protein 1 beta; sCD40L, Soluble CD40 ligand; VEGF, vascular endothelial growth factor; and TGF-α, Transforming growth factor alpha CLL, chronic lymphocytic leukemia; DLBCL, diffuse large B-cell lymphoma; FL, follicular lymphoma; MM, multiple myeloma; Models were adjusted for age, gender, country, body mass index, education, physical activity, smoking status at enrolment, and alcohol intake at enrolment .

**Supplementary Table 7-B.** **Results of the mixed model relating log-transformed concentrations of immune markers and all B-cell lymphoma cases and main histological subtypes for samples from Phase 2 (172 pairs).**

|  | **All BCL** | |  | **CLL** | |  | **DLBCL** | |  | **FL** | |  | **MM** | |
| --- | --- | --- | --- | --- | --- | --- | --- | --- | --- | --- | --- | --- | --- | --- |
|  | *β* | *p-value* |  | *β* | *p-value* |  | *β* | *p-value* |  | *β* | *p-value* |  | *β* | *p-value* |
| EGF | -0.11 | 5.45e-01 |  | -0.03 | 9.17e-01 |  | -0.34 | 3.43e-01 |  | 0.11 | 7.83e-01 |  | -0.32 | 2.61e-01 |
| Eotaxin | 0.06 | 2.38e-01 |  | 0.23 | 8.60e-03 |  | 0.23 | 2.79e-02 |  | 0.07 | 5.16e-01 |  | 0.02 | 7.29e-01 |
| FGF2 | -0.41 | 3.08e-02 |  | -0.13 | 7.15e-01 |  | 0.05 | 9.05e-01 |  | 0.01 | 9.75e-01 |  | -1.05 | 2.82e-04 |
| Fractalkine | -0.37 | 4.89e-02 |  | -0.24 | 4.74e-01 |  | 0.05 | 8.84e-01 |  | -0.12 | 7.34e-01 |  | -0.83 | 5.07e-03 |
| GCSF | -0.19 | 1.14e-01 |  | -0.15 | 5.17e-01 |  | -0.21 | 3.90e-01 |  | -0.04 | 8.34e-01 |  | -0.24 | 2.11e-01 |
| GRO | 0.03 | 5.74e-01 |  | -0.01 | 9.24e-01 |  | 0.11 | 3.23e-01 |  | -0.08 | 5.08e-01 |  | 0.08 | 3.15e-01 |
| INFa | -0.28 | 2.43e-01 |  | -0.2 | 6.39e-01 |  | 0.23 | 6.49e-01 |  | -0.06 | 9.00e-01 |  | -0.2 | 5.96e-01 |
| IP10 | -0.03 | 7.03e-01 |  | 0.13 | 3.22e-01 |  | -0.13 | 3.90e-01 |  | -0.07 | 6.71e-01 |  | -0.06 | 6.36e-01 |
| MCP1 | -0.04 | 5.84e-01 |  | 0.18 | 1.84e-01 |  | 0.07 | 6.85e-01 |  | -0.22 | 2.16e-01 |  | -0.08 | 5.57e-01 |
| MCP3 | -0.3 | 1.51e-01 |  | -0.35 | 3.28e-01 |  | 0.05 | 9.01e-01 |  | -0.02 | 9.10e-01 |  | -0.79 | 1.37e-02 |
| MDC | -0.13 | 3.86e-02 |  | 0 | 9.89e-01 |  | -0.17 | 1.98e-01 |  | -0.04 | 7.58e-01 |  | -0.15 | 8.38e-02 |
| MIP1a | -0.2 | 2.39e-01 |  | 0.42 | 1.92e-01 |  | 0.09 | 8.29e-01 |  | -0.29 | 4.58e-01 |  | -0.63 | 2.35e-02 |
| MIP1b | -0.17 | 2.00e-01 |  | 0.11 | 6.44e-01 |  | -0.12 | 6.67e-01 |  | -0.06 | 8.47e-01 |  | -0.42 | 5.10e-02 |
| sCD40L | 0.16 | 7.72e-02 |  | 0.28 | 6.54e-02 |  | 0.17 | 3.29e-01 |  | 0.2 | 2.75e-01 |  | 0.12 | 3.44e-01 |
| TGFa | -0.44 | 3.80e-02 |  | -0.45 | 2.28e-01 |  | -0.21 | 6.47e-01 |  | -0.23 | 6.13e-01 |  | -0.89 | 6.04e-03 |
| VEGF | -0.38 | 5.92e-02 |  | 0.36 | 3.23e-01 |  | -0.08 | 8.45e-01 |  | -0.16 | 7.12e-01 |  | -1.07 | 6.20e-04 |
| IL1b | -0.14 | 2.86e-01 |  | -0.49 | 3.18e-02 |  | 0.26 | 3.50e-01 |  | 0.1 | 7.39e-01 |  | 0.02 | 9.17e-01 |
| IL2 | -0.1 | 4.65e-01 |  | -0.38 | 1.32e-01 |  | 0.33 | 2.66e-01 |  | 0.03 | 9.29e-01 |  | 0.13 | 5.41e-01 |
| IL4 | -0.06 | 7.06e-01 |  | -0.68 | 1.80e-02 |  | 0.08 | 8.10e-01 |  | 0.14 | 6.90e-01 |  | 0.26 | 2.99e-01 |
| IL5 | -0.1 | 3.74e-01 |  | -0.34 | 8.05e-02 |  | 0.01 | 9.63e-01 |  | -0.06 | 7.88e-01 |  | 0.17 | 3.37e-01 |
| IL6 | -0.04 | 7.30e-01 |  | -0.24 | 2.27e-01 |  | -0.04 | 8.75e-01 |  | -0.25 | 2.82e-01 |  | 0.07 | 6.63e-01 |
| IL7 | 0.05 | 7.25e-01 |  | -0.24 | 3.80e-01 |  | 0.12 | 7.21e-01 |  | 0.13 | 7.28e-01 |  | 0.15 | 5.22e-01 |
| IL8 | -0.15 | 3.55e-02 |  | -0.28 | 1.56e-02 |  | -0.02 | 9.08e-01 |  | -0.16 | 2.86e-01 |  | -0.08 | 4.76e-01 |
| IL10 | 0.07 | 5.07e-01 |  | -0.12 | 5.82e-01 |  | 0.18 | 4.81e-01 |  | 0.29 | 2.81e-01 |  | 0.15 | 4.10e-01 |
| IL13 | -0.09 | 4.89e-01 |  | -0.27 | 2.60e-01 |  | -0.14 | 6.20e-01 |  | -0.15 | 6.24e-01 |  | 0.15 | 5.12e-01 |
| INFg | -0.21 | 2.24e-01 |  | -0.55 | 6.42e-02 |  | 0 | 9.91e-01 |  | 0.23 | 5.49e-01 |  | -0.12 | 6.54e-01 |
| GMCSF | -0.12 | 4.46e-01 |  | -0.55 | 4.47e-02 |  | 0.26 | 4.39e-01 |  | 0.06 | 8.74e-01 |  | 0.05 | 8.27e-01 |
| TNFa | 0.12 | 1.46e-01 |  | 0.07 | 6.55e-01 |  | 0.13 | 4.89e-01 |  | 0.11 | 5.80e-01 |  | 0.29 | 2.34e-02 |

IL indicates Interleukin; INF-α, interferon alpha; INF-γ, interferon gamma; GMCSF, Granulocyte-macrophage colony stimulating factor; TNF-α, Tumor necrosis factor alpha; EGF, Epidermal growth factor; FGF-2, Fibroblast growth factor 2; GCSF, Granulocyte colony-stimulating factor; GRO, melanoma growth stimulatory activity/growth-related oncogene; IP10, Interferon gamma-induced protein 10; MCP-1, Monocyte chemotactic protein-1; MCP-3, Monocyte chemotactic protein-3; MDC, Macrophage Derived Chemokine; MIP-1α, Macrophage Inflammatory Protein 1 alpha; MIP-1ß, Macrophage Inflammatory Protein 1 beta; sCD40L, Soluble CD40 ligand; VEGF, vascular endothelial growth factor; and TGF-α, Transforming growth factor alpha CLL, chronic lymphocytic leukemia; DLBCL, diffuse large B-cell lymphoma; FL, follicular lymphoma; MM, multiple myeloma; ; Models were adjusted for age, gender, country, body mass index, education, physical activity, smoking status at enrolment, and alcohol intake at enrolment .

**Supplementary Table 8.** **Results of the mixed model relating log-transformed concentrations of immune markers and all B-cell lymphoma. Results are presented for cases diagnosed less than 6 years after blood collection.**

|  | **All BCL** | |  | **CLL** | | |  | | **DLBCL** | | | |  | | **MM** | | | |  |
| --- | --- | --- | --- | --- | --- | --- | --- | --- | --- | --- | --- | --- | --- | --- | --- | --- | --- | --- | --- |
|  | *β* | *p-value* |  | *β* | *p-value* | |  | | *β* | | *p-value* | |  | | *β* | | *p-value* | |  |
| EGF | -0.18 | 5.94e-01 |  | -0.14 | | 7.27e-01 | |  | | -0.93 | | 1.91e-02 | |  | | -0.74 | | 1.05e-02 | |
| Eotaxin | -0.04 | 6.88e-01 |  | 0.09 | | 5.16e-01 | |  | | -0.01 | | 9.36e-01 | |  | | 0.01 | | 8.91e-01 | |
| FGF2 | -1.01 | 2.17e-03 |  | -0.36 | | 4.28e-01 | |  | | -0.53 | | 1.97e-01 | |  | | -1.15 | | 1.08e-04 | |
| Fractalkine | -0.58 | 1.13e-01 |  | -0.21 | | 5.96e-01 | |  | | -0.46 | | 2.35e-01 | |  | | -0.91 | | 4.51e-04 | |
| GCSF | -0.08 | 7.67e-01 |  | -0.19 | | 4.56e-01 | |  | | -0.28 | | 2.47e-01 | |  | | -0.15 | | 3.81e-01 | |
| GRO | -0.01 | 9.26e-01 |  | 0 | | 9.88e-01 | |  | | 0.03 | | 8.32e-01 | |  | | 0.02 | | 7.95e-01 | |
| INFa | -0.06 | 9.01e-01 |  | 0.22 | | 7.68e-01 | |  | | -1.02 | | 1.60e-01 | |  | | -1.01 | | 5.43e-02 | |
| IP10 | 0.03 | 8.58e-01 |  | 0.01 | | 9.42e-01 | |  | | 0.08 | | 4.83e-01 | |  | | -0.01 | | 9.23e-01 | |
| MCP1 | 0.16 | 3.13e-01 |  | -0.01 | | 8.88e-01 | |  | | -0.05 | | 5.94e-01 | |  | | -0.07 | | 4.28e-01 | |
| MCP3 | -0.81 | 2.46e-02 |  | -0.32 | | 5.10e-01 | |  | | -0.58 | | 1.91e-01 | |  | | -0.93 | | 2.87e-03 | |
| MDC | -0.09 | 4.34e-01 |  | -0.14 | | 4.53e-01 | |  | | -0.37 | | 1.73e-02 | |  | | -0.27 | | 1.10e-02 | |
| MIP1a | -0.22 | 5.44e-01 |  | 0.41 | | 2.69e-01 | |  | | -0.59 | | 9.02e-02 | |  | | -1.07 | | 3.01e-05 | |
| MIP1b | -0.33 | 1.94e-01 |  | 0.25 | | 4.20e-01 | |  | | -0.3 | | 2.78e-01 | |  | | -0.56 | | 8.47e-03 | |
| sCD40L | 0.29 | 1.13e-01 |  | 0.13 | | 4.86e-01 | |  | | 0.28 | | 9.85e-02 | |  | | -0.05 | | 7.10e-01 | |
| TGFa | -0.71 | 7.14e-02 |  | -0.2 | | 7.00e-01 | |  | | -0.87 | | 7.19e-02 | |  | | -1.26 | | 3.03e-04 | |
| VEGF | -0.79 | 2.97e-02 |  | -0.08 | | 8.64e-01 | |  | | -0.67 | | 1.03e-01 | |  | | -1.22 | | 1.18e-04 | |
| IL1b | -0.76 | 1.70e-02 |  | 0.11 | | 8.29e-01 | |  | | -0.12 | | 7.83e-01 | |  | | -0.62 | | 2.81e-02 | |
| IL2 | -0.07 | 8.03e-01 |  | 0.02 | | 9.55e-01 | |  | | 0 | | 9.93e-01 | |  | | -0.37 | | 1.55e-01 | |
| IL4 | 0.05 | 8.87e-01 |  | -1.01 | | 2.30e-02 | |  | | -0.38 | | 3.70e-01 | |  | | -0.08 | | 7.80e-01 | |
| IL5 | -0.25 | 3.36e-01 |  | -0.05 | | 8.69e-01 | |  | | -0.01 | | 9.75e-01 | |  | | -0.07 | | 7.28e-01 | |
| IL6 | -0.14 | 6.09e-01 |  | -0.39 | | 2.56e-01 | |  | | -0.51 | | 1.29e-01 | |  | | -0.34 | | 1.40e-01 | |
| IL7 | 0.03 | 9.19e-01 |  | -0.58 | | 1.14e-01 | |  | | 0.04 | | 9.52e-01 | |  | | -0.07 | | 8.14e-01 | |
| IL8 | 0.01 | 9.63e-01 |  | -0.04 | | 8.40e-01 | |  | | 0.03 | | 8.73e-01 | |  | | -0.22 | | 9.89e-02 | |
| IL10 | 0.2 | 4.57e-01 |  | -0.2 | | 5.59e-01 | |  | | -0.08 | | 7.60e-01 | |  | | -0.17 | | 4.94e-01 | |
| IL13 | -0.41 | 2.68e-01 |  | 0.26 | | 6.27e-01 | |  | | -0.2 | | 6.68e-01 | |  | | -0.7 | | 3.62e-02 | |
| INFg | -0.86 | 3.79e-02 |  | -0.19 | | 7.21e-01 | |  | | -0.27 | | 6.07e-01 | |  | | -0.71 | | 5.07e-02 | |
| GMCSF | -0.47 | 1.20e-01 |  | 0.24 | | 6.01e-01 | |  | | 0.46 | | 2.42e-01 | |  | | -0.06 | | 8.19e-01 | |
| TNFa | 0.3 | 2.20e-02 |  | 0 | | 9.96e-01 | |  | | 0.23 | | 2.39e-01 | |  | | -0.05 | | 7.08e-01 | |

IL indicates Interleukin; INF-α, interferon alpha; INF-γ, interferon gamma; GMCSF, Granulocyte-macrophage colony stimulating factor; TNF-α, Tumor necrosis factor alpha; EGF, Epidermal growth factor; FGF-2, Fibroblast growth factor 2; GCSF, Granulocyte colony-stimulating factor; GRO, melanoma growth stimulatory activity/growth-related oncogene; IP10, Interferon gamma-induced protein 10; MCP-1, Monocyte chemotactic protein-1; MCP-3, Monocyte chemotactic protein-3; MDC, Macrophage Derived Chemokine; MIP-1α, Macrophage Inflammatory Protein 1 alpha; MIP-1ß, Macrophage Inflammatory Protein 1 beta; sCD40L, Soluble CD40 ligand; VEGF, vascular endothelial growth factor; and TGF-α, Transforming growth factor alpha CLL, chronic lymphocytic leukemia; DLBCL, diffuse large B-cell lymphoma; FL, follicular lymphoma; MM, multiple myeloma; ; Models were adjusted for age, gender, country, experimental phase, body mass index, education, physical activity, smoking status at enrolment, and alcohol intake at enrolment .

**Supplementary Table 9.** **Results of the mixed model relating log-transformed concentrations of immune markers and all B-cell lymphoma. Results are presented for cases diagnosed more than 6 years after blood collection.**

|  | **All BCL** | |  | **CLL** | | |  | | **DLBCL** | | | |  | | **MM** | | | |  |
| --- | --- | --- | --- | --- | --- | --- | --- | --- | --- | --- | --- | --- | --- | --- | --- | --- | --- | --- | --- |
|  | *β* | *p-value* |  | *β* | *p-value* | |  | | *β* | | *p-value* | |  | | *β* | | *p-value* | |  |
| EGF | -0.74 | 1.05e-02 |  | 0.32 | | 3.80e-01 | |  | | -0.32 | | 3.89e-01 | |  | | -0.18 | | 5.94e-01 | |
| Eotaxin | 0.01 | 8.91e-01 |  | 0.23 | | 1.12e-02 | |  | | 0.21 | | 3.34e-02 | |  | | -0.04 | | 6.88e-01 | |
| FGF2 | -1.15 | 1.08e-04 |  | 0.14 | | 6.81e-01 | |  | | 0.11 | | 7.53e-01 | |  | | -1.01 | | 2.17e-03 | |
| Fractalkine | -0.91 | 4.51e-04 |  | 0.26 | | 4.85e-01 | |  | | 0.19 | | 6.21e-01 | |  | | -0.58 | | 1.13e-01 | |
| GCSF | -0.15 | 3.81e-01 |  | 0.04 | | 8.88e-01 | |  | | -0.02 | | 9.46e-01 | |  | | -0.08 | | 7.67e-01 | |
| GRO | 0.02 | 7.95e-01 |  | 0.1 | | 4.04e-01 | |  | | 0.11 | | 3.37e-01 | |  | | -0.01 | | 9.26e-01 | |
| INFa | -1.01 | 5.43e-02 |  | -0.25 | | 6.52e-01 | |  | | -1.01 | | 8.92e-02 | |  | | -0.06 | | 9.01e-01 | |
| IP10 | -0.01 | 9.23e-01 |  | 0.27 | | 7.58e-02 | |  | | 0.03 | | 8.30e-01 | |  | | 0.03 | | 8.58e-01 | |
| MCP1 | -0.07 | 4.28e-01 |  | 0.32 | | 5.56e-02 | |  | | 0.07 | | 7.12e-01 | |  | | 0.16 | | 3.13e-01 | |
| MCP3 | -0.93 | 2.87e-03 |  | -0.05 | | 9.24e-01 | |  | | 0.32 | | 4.33e-01 | |  | | -0.81 | | 2.46e-02 | |
| MDC | -0.27 | 1.10e-02 |  | 0.06 | | 6.12e-01 | |  | | -0.19 | | 2.24e-01 | |  | | -0.09 | | 4.34e-01 | |
| MIP1a | -1.07 | 3.01e-05 |  | 0.19 | | 5.72e-01 | |  | | 0.01 | | 9.86e-01 | |  | | -0.22 | | 5.44e-01 | |
| MIP1b | -0.56 | 8.47e-03 |  | 0.14 | | 5.99e-01 | |  | | -0.1 | | 7.25e-01 | |  | | -0.33 | | 1.94e-01 | |
| sCD40L | -0.05 | 7.10e-01 |  | 0.59 | | 4.24e-03 | |  | | 0.12 | | 5.85e-01 | |  | | 0.29 | | 1.13e-01 | |
| TGFa | -1.26 | 3.03e-04 |  | -0.62 | | 1.20e-01 | |  | | -0.44 | | 3.26e-01 | |  | | -0.71 | | 7.14e-02 | |
| VEGF | -1.22 | 1.18e-04 |  | 0.61 | | 1.14e-01 | |  | | -0.18 | | 6.65e-01 | |  | | -0.79 | | 2.97e-02 | |
| IL1b | -0.62 | 2.81e-02 |  | -0.68 | | 4.12e-02 | |  | | 0.71 | | 3.48e-02 | |  | | -0.76 | | 1.70e-02 | |
| IL2 | -0.37 | 1.55e-01 |  | -0.07 | | 8.24e-01 | |  | | 0.06 | | 8.57e-01 | |  | | -0.07 | | 8.03e-01 | |
| IL4 | -0.08 | 7.80e-01 |  | -0.6 | | 9.37e-02 | |  | | -0.2 | | 6.09e-01 | |  | | 0.05 | | 8.87e-01 | |
| IL5 | -0.07 | 7.28e-01 |  | -0.19 | | 4.96e-01 | |  | | 0.34 | | 2.33e-01 | |  | | -0.25 | | 3.36e-01 | |
| IL6 | -0.34 | 1.40e-01 |  | 0.13 | | 6.42e-01 | |  | | 0.08 | | 7.82e-01 | |  | | -0.14 | | 6.09e-01 | |
| IL7 | -0.07 | 8.14e-01 |  | 0.11 | | 7.30e-01 | |  | | 0.03 | | 9.17e-01 | |  | | 0.03 | | 9.19e-01 | |
| IL8 | -0.22 | 9.89e-02 |  | -0.08 | | 6.19e-01 | |  | | 0.02 | | 9.16e-01 | |  | | 0.01 | | 9.63e-01 | |
| IL10 | -0.17 | 4.94e-01 |  | 0.16 | | 5.90e-01 | |  | | 0.31 | | 3.10e-01 | |  | | 0.2 | | 4.57e-01 | |
| IL13 | -0.7 | 3.62e-02 |  | -0.09 | | 8.09e-01 | |  | | 0.66 | | 9.64e-02 | |  | | -0.41 | | 2.68e-01 | |
| INFg | -0.71 | 5.07e-02 |  | 0.13 | | 7.74e-01 | |  | | 0.19 | | 6.35e-01 | |  | | -0.86 | | 3.79e-02 | |
| GMCSF | -0.06 | 8.19e-01 |  | -0.48 | | 1.23e-01 | |  | | 0.03 | | 9.10e-01 | |  | | -0.47 | | 1.20e-01 | |
| TNFa | -0.05 | 7.08e-01 |  | 0.32 | | 3.17e-02 | |  | | -0.03 | | 8.36e-01 | |  | | 0.3 | | 2.20e-02 | |

IL indicates Interleukin; INF-α, interferon alpha; INF-γ, interferon gamma; GMCSF, Granulocyte-macrophage colony stimulating factor; TNF-α, Tumor necrosis factor alpha; EGF, Epidermal growth factor; FGF-2, Fibroblast growth factor 2; GCSF, Granulocyte colony-stimulating factor; GRO, melanoma growth stimulatory activity/growth-related oncogene; IP10, Interferon gamma-induced protein 10; MCP-1, Monocyte chemotactic protein-1; MCP-3, Monocyte chemotactic protein-3; MDC, Macrophage Derived Chemokine; MIP-1α, Macrophage Inflammatory Protein 1 alpha; MIP-1ß, Macrophage Inflammatory Protein 1 beta; sCD40L, Soluble CD40 ligand; VEGF, vascular endothelial growth factor; and TGF-α, Transforming growth factor alpha CLL, chronic lymphocytic leukemia; DLBCL, diffuse large B-cell lymphoma; FL, follicular lymphoma; MM, multiple myeloma; ; Models were adjusted for age, gender, country, experimental phase, body mass index, education, physical activity, smoking status at enrolment, and alcohol intake at enrolment .

**Supplementary Table 10.** Conditional logistic regression comparing MM cases to individually age- and sex-matched controls

|  |  | Ca/Co, *n* | OR (95% CI) |
| --- | --- | --- | --- |
| FGF-2 | Q1 (<1.76) | 33/21 | Ref. |
|  | Q2 (1.76-3.25) | 23/15 | 1.88 (0.54-6.60) |
|  | Q3 (3.26-4.37) | 15/25 | 0.36 (0.13-0.98) |
|  | Q4 (>4.37) | 5/15 | 0.21 (0.05-0.98) |
|  | *P***-**trend |  | 0.008 |
| Fractalkine | Q1 (<2.6) | 32/18 | Ref. |
|  | Q2 (2.6-4.00) | 18/20 | 0.51 (0.20-1.28) |
|  | Q3 (4.01-5.10) | 19/22 | 0.42 (0.15-1.21) |
|  | Q4 (>5.10) | 7/16 | 0.22 (0.06-0.85) |
|  | *P***-**trend |  | 0.02 |
| MCP-3 | Q1 (<0.57) | 29/18 | Ref. |
|  | Q2 (0.57-2.59) | 27/25 | 0.73 (0.28-1.92) |
|  | Q3 (2.60-3.67) | 11/15 | 0.43 (0.14-1.32) |
|  | Q4 (>3.67) | 9/18 | 0.45 (0.15-1.33) |
|  | *P***-**trend |  | 0.08 |
| MIP-1α | Q1 (<0.56) | 21/18 | Ref. |
|  | Q2 (0.56-2.48) | 29/20 | 1.84 (0.61-5.60) |
|  | Q3 (2.49-3.72) | 19/19 | 0.67 (0.21-2.09) |
|  | Q4 (>3.72) | 7/19 | 0.30 (0.07-1.24) |
|  | *P***-**trend |  | 0.09 |
| TGF-α | Q1 (<-0.57) | 28/15 | Ref. |
|  | Q2 (-0.57-0.79) | 28/24 | 0.42 (0.15-1.18) |
|  | Q3 (0.80-2.62) | 11/19 | 0.19 (0.05-0.71) |
|  | Q4 (>2.62) | 9/18 | 0.10 (0.02-0.53) |
|  | *P***-**trend |  | 0.003 |
| VEGF | Q1 (<3.4) |  | Ref. |
|  | Q2 (3.4-5.29) | 31/19 | 0.48 (0.16-1.46) |
|  | Q3 (5.3-6.56) | 21/21 | 0.37 (0.13-1.02) |
|  | Q4 (>6.56) | 14/21 | 0.50 (0.15-1.60) |
|  | *P***-**trend | 10/15 | 0.11 |

Models were adjusted for experimental phase, body mass index, education, physical activity, smoking status at enrolment, and alcohol intake at enrolment.

**Supplementary Figure 1.** **Results of the mixed model analyses between log-transformed values of immune markers and all B-cell lymphoma cases excluding Multiple Myeloma cases.** Strength of association (Y-axis) is measured by p-values and the black horizontal line represents the Bonferroni cut-off value ensuring a control of the family wise error rate (FWER) below 0.05. Inverse associations are represented in blue and positive associations in orange. Models were adjusted for age, gender, country, experimental phase, body mass index, education, physical activity, smoking status at enrolment, and alcohol intake at enrolment. Results are presented for the full study population (triangles, N=192 case/control pairs), and for the (N=157) pairs in which white blood cell composition estimates were available, models are either adjusted (squares) or unadjusted (diamonds) of WBC proportions.

IL indicates Interleukin; INF-α, interferon alpha; INF-γ, interferon gamma; GMCSF, Granulocyte-macrophage colony stimulating factor; TNF-α, Tumor necrosis factor alpha; EGF, Epidermal growth factor; FGF-2, Fibroblast growth factor 2; GCSF, Granulocyte colony-stimulating factor; GRO, melanoma growth stimulatory activity/growth-related oncogene; IP10, Interferon gamma-induced protein 10; MCP-1, Monocyte chemotactic protein-1; MCP-3, Monocyte chemotactic protein-3; MDC, Macrophage Derived Chemokine; MIP-1α, Macrophage Inflammatory Protein 1 alpha; MIP-1ß, Macrophage Inflammatory Protein 1 beta; sCD40L, Soluble CD40 ligand; VEGF, vascular endothelial growth factor; and TGF-α, Transforming growth factor alpha.

**Supplementary Figure 2.** **Stability analysis of subtype specific sPLS-DA models.** For each protein (X-axis) and each possible number of selected variables (Y-axis), the selection proportion across the 10,000 repeats subsamples are reported and color coded. Results are presented for DLBCL (A), CLL (B), and MM (C)

IL indicates Interleukin; INF-α, interferon alpha; INF-γ, interferon gamma; GMCSF, Granulocyte-macrophage colony stimulating factor; TNF-α, Tumor necrosis factor alpha; EGF, Epidermal growth factor; FGF-2, Fibroblast growth factor 2; GCSF, Granulocyte colony-stimulating factor; GRO, melanoma growth stimulatory activity/growth-related oncogene; IP10, Interferon gamma-induced protein 10; MCP-1, Monocyte chemotactic protein-1; MCP-3, Monocyte chemotactic protein-3; MDC, Macrophage Derived Chemokine; MIP-1α, Macrophage Inflammatory Protein 1 alpha; MIP-1ß, Macrophage Inflammatory Protein 1 beta; sCD40L, Soluble CD40 ligand; VEGF, vascular endothelial growth factor; and TGF-α, Transforming growth factor alpha.
